# The Influence of Study-Level Inference Models and Study Set Size on Coordinate-Based fMRI Meta-Analyses

**DOI:** 10.3389/fnins.2017.00745

**Published:** 2018-01-18

**Authors:** Han Bossier, Ruth Seurinck, Simone Kühn, Tobias Banaschewski, Gareth J. Barker, Arun L. W. Bokde, Jean-Luc Martinot, Herve Lemaitre, Tomáš Paus, Sabina Millenet, Beatrijs Moerkerke

**Affiliations:** ^1^Department of Data Analysis, Ghent University, Ghent, Belgium; ^2^Department of Psychiatry and Psychotherapy, University Clinic, Hamburg-Eppendorf, Germany; ^3^Department of Child and Adolescent Psychiatry and Psychotherapy, Central Institute of Mental Health, Medical Faculty Mannheim, Heidelberg University, Mannheim, Germany; ^4^Department of Neuroimaging, Institute of Psychiatry, Psychology & Neuroscience, King's College London, London, United Kingdom; ^5^Discipline of Psychiatry, School of Medicine and Trinity College Institute of Neuroscience, Trinity College Dublin, Dublin, Ireland; ^6^Institut National de la Santé et de la Recherche Médicale, INSERM Unit 1000 Neuroimaging & Psychiatry, University Paris Sud – Paris Saclay, University Paris Descartes; and Maison de Solenn, Paris, France; ^7^Institut National de la Santé et de la Recherche Médicale, INSERM Unit 1000 “Neuroimaging & Psychiatry”, Faculté de médecine, Université Paris-Sud, Le Kremlin-Bicêtre; and Université Paris Descartes, Sorbonne Paris Cité, Paris, France; ^8^Baycrest and Departments of Psychology and Psychiatry, Rotman Research Institute, University of Toronto, Toronto, ON, Canada

**Keywords:** coordinate-based meta-analysis, fMRI, group modeling, mixed effects models, random effects models, reliability

## Abstract

Given the increasing amount of neuroimaging studies, there is a growing need to summarize published results. Coordinate-based meta-analyses use the locations of statistically significant local maxima with possibly the associated effect sizes to aggregate studies. In this paper, we investigate the influence of key characteristics of a coordinate-based meta-analysis on (1) the balance between false and true positives and (2) the activation reliability of the outcome from a coordinate-based meta-analysis. More particularly, we consider the influence of the chosen group level model at the study level [fixed effects, ordinary least squares (OLS), or mixed effects models], the type of coordinate-based meta-analysis [Activation Likelihood Estimation (ALE) that only uses peak locations, fixed effects, and random effects meta-analysis that take into account both peak location and height] and the amount of studies included in the analysis (from 10 to 35). To do this, we apply a resampling scheme on a large dataset (*N* = 1,400) to create a test condition and compare this with an independent evaluation condition. The test condition corresponds to subsampling participants into studies and combine these using meta-analyses. The evaluation condition corresponds to a high-powered group analysis. We observe the best performance when using mixed effects models in individual studies combined with a random effects meta-analysis. Moreover the performance increases with the number of studies included in the meta-analysis. When peak height is not taken into consideration, we show that the popular ALE procedure is a good alternative in terms of the balance between type I and II errors. However, it requires more studies compared to other procedures in terms of activation reliability. Finally, we discuss the differences, interpretations, and limitations of our results.

## Introduction

Over the past two decades, there has been a substantial increase in the number of functional Magnetic Resonance Imaging (fMRI) studies, going from 20 publications in 1994 to over 5000 in 2015. Despite this vast amount of fMRI literature, it remains challenging to establish scientific truth.

First, fMRI studies tend to have small sample sizes to detect realistic effect sizes (median estimated sample size in 2015 = 28.5; Poldrack et al., 2017) as among other causes scanning participants is costly and time consuming. The large multiple testing problem and ensuing corrections make statistical testing in fMRI conservative, thereby further reducing statistical power or probability to detect true activation (Lieberman and Cunningham, 2009; Durnez et al., 2014). As a consequence, the probability that a statistically significant effect reflects true activation is reduced (Button et al., 2013). This can lead to more false negatives (missing true activation) as well as more false positives (detecting activation where there is none) in published fMRI studies. Second, the diversity of pre-processing steps and analysis pipelines have made fMRI studies challenging to replicate (Carp, 2012a,b) even though researchers recognize the value of both reproducibility (obtaining identical parameter estimates compared to the original experiment using the same analysis and data; Poldrack and Poline, 2015) and replicability (the ability of an entire experiment to be replicated by gathering new data using the exact same materials and methods; Patil et al., 2016). Roels et al. (2015) also showed there is variability in the number of significant features (i.e., peaks or clusters of activity) depending on the data-analytical methods used. Several approaches have been offered to overcome these challenges. A first remediating step is to promote transparency, pre-registration and open science initiatives such as data sharing or using standardized protocols in organizing and managing data (Poline et al., 2012; Pernet and Poline, 2015; Gorgolewski and Poldrack, 2016; Gorgolewski et al., 2016; Poldrack et al., 2017). A second approach to establish scientific truth across studies, is to accumulate knowledge by scientifically combining previous results using meta-analysis (Lieberman and Cunningham, 2009; Yarkoni et al., 2010). Combining findings across studies increases power to detect true effects, while false positives are not expected to replicate across studies, given a representative set of unbiased results. Furthermore, meta-analyses can generate new scientific questions (Wager et al., 2009).

Originally, meta-analyses were developed to aggregate single univariate effect sizes (Borenstein et al., 2009). In an individual fMRI study however, the brain is divided in a large amount of artificially created cubes (voxels). Until recently, the standard approach was to only report coordinates in 3D space of peaks of activity that survive a statistical threshold. These are called foci, peaks, or local maxima. While guidelines are shifting toward making statistical maps or full data sets of a study available, many findings in the literature only consist of locations of activation. In these cases, an fMRI meta-analysis is limited to those voxels for which information is at hand. This is termed a coordinate-based meta-analysis (CBMA, see e.g., Paus, 1996; Paus et al., 1998). When full images (and hence information in all voxels) are available, methods designed for image-based meta-analysis (IBMA) can be used (Salimi-Khorshidi et al., 2009; Radua and Mataix-Cols, 2012).

In this study, we focus on CBMA for which different algorithms exist (Wager et al., 2007; Radua et al., 2012). In particular, we consider the popular Activation Likelihood Estimation (ALE) (Turkeltaub et al., 2002, 2012) and effect size based methods such as seed based *d*-mapping (SBdM, formerly called effect size-signed differential mapping, RRID:SCR_002554) (Radua et al., 2012). The ALE algorithm considers a reported local maximum as a center of a spatial probability distribution. As such, the method only requires the location of the peak and then searches for brain regions where spatial convergence can be distinguished from random clustering of peaks. Effect size based methods on the other hand transform *t*-values of reported local maxima into effect size estimates and calculate a weighted average of the reported evidence. The weights determine the underlying meta-analysis model. For instance, the weights in seed based *d*-mapping include within-study and between-study variability which corresponds to a random effects model. If the weights ignore the between-study variability one obtains a fixed effects model.

In this paper, we evaluate the influence of study characteristics on the statistical properties of CBMA techniques for fMRI. Previous work by Eickhoff et al. (2016a) and Radua et al. (2012) already evaluated statistical properties of CBMA algorithms or tested software for implementation errors (Eickhoff et al., 2016b). However, these studies did not study the effect of input characteristics at the individual study level on the performance of these CBMA algorithms. We investigate the influence of the group level model on the performance of various CBMA procedures. More specifically, we test the effect of pooling subjects at the individual study level using either a fixed effects, ordinary least squares (OLS) or mixed effects group level model on the outcome of the meta-analyses methods mentioned above. As in Eickhoff et al. (2016a) we also evaluate the effect of the number of studies in the meta-analysis (*K*). Extending on their work, we consider the case for *K* = 10, 12, 14, 16, 18, 20, 30, and 35 when using ALE as well as effect size based CBMA's using a fixed and random effects model. We consider two performance measures: the balance between false positives and true positives and the activation reliability as a proxy for replicability.

We approach this problem by applying a resampling scheme on a large dataset from the IMAGEN project (Schumann et al., 2010) and create meta-analyses (i.e., test conditions) which we compare against a high powered large sample size study as a reference (i.e., an evaluation condition).

In the following section, we discuss the dataset, give a theoretical overview of the three models to pool subjects at study level and discuss the three models for coordinate-based meta-analysis. In the sections thereafter, we present the design of the study with the chosen performance measures and discuss our findings.

## Materials and methods

The code containing the design and analysis of the results in this paper are available at: https://github.com/NeuroStat/PaperStudyCharCBMA.

### Data

We use preprocessed data from the IMAGEN project (Schumann et al., 2010). This is a large genetic-neuroimaging study on reinforcement-related behavior in adolescents with the goal to identify its predictive value for the development of frequent psychiatric disorders across Europe. The database contains fMRI data from 1,487 adolescents aged between 13 and 15 years, acquired across several research centers on 3 Tesla scanners from different manufactures. The data are stored and preprocessed at the Neurospin^1^ center (France) using SPM8^2^ (Statistical Parametric Mapping: Wellcome Department of Cognitive Neurology, London, UK).

The scanning sessions of interest involved a global cognitive assessment. In a fast-event related design, participants had to do a series of alternating cognitive/motor tasks. While the total series contains 10 types of tasks, we restrict our analysis to one contrast of two types. These are (1) reading sentences in silence and (2) solving math subtractions in silence. The math questions were single digits (0–9) that had to be subtracted from a digit between 11 and 20. Each of these two type of trials were presented for 10 times with a probabilistic inter-stimulus interval of on average 3 s (see also Pinel et al., 2007). We use the contrast MATH > LANGUAGE (2–1) for this study.

A BOLD time series was recorded for each participant using echoplanar imaging with an isotropic voxel size of 3.4 mm, and temporal resolutions of 2.2 s. A total of 160 volumes were obtained. For each participant, a structural T1-weighted image (based on the ADNI protocols^3^ was acquired for registration.

Preprocessing included slice-timing correction, movement correction, coregistration to the segmented structural T1-weighted images, non-linear warping on the MNI space using a custom EPI template and spatial smoothing of the signal with a 5 mm Gaussian Kernel (Imagen fMRI data analysis methods, revision2, July 2010).

In the first level analysis, all experimental manipulations were modeled using a general linear model (GLM) with a standard autoregressive [AR(1)] noise model and 18 estimated movement parameters as nuisance terms. This resulted in a statistical map for each parameter estimate and a map reflecting the residual variance of the model fit. In this study, we use for each participant (1) the contrast map or the difference between the parameter estimate maps for MATH and LANGUAGE and (2) a variance (squared standard error) image for that contrast derived from the residual variance map. After visual inspection for errors or artifacts we removed 87 participants from which parts of the brain were missing. To automate, we used a cut-off corresponding to 96% of the median number of masked voxels over all subjects in the database.

### Group level models

Localizing significant brain activity in an fMRI data-analysis is based on the statistical parametric map of contrasting conditions associated with all participants involved in an experiment. In this study, we focus on the univariate approach in which activation is tested in a voxelwise manner through GLMs. Due to computational constraints, the analysis is typically executed in a two stage GLM procedure (Beckmann et al., 2003). In a first step, the measured time series (BOLD signal) of each subject is modeled by a linear combination of nuisance terms and the expected time series under the experimental design (Friston et al., 1994). Note that such a model is fitted for each voxel v (v = 1, …, *S*) separately. In a second step, parameter estimates obtained at the first stage are combined over *N* subjects to obtain group level estimates. More particularly, we use the vector of estimated first level contrasts ${\text{Y}}_{\text{G}}\;=\;{\left[\text{c}\hat{\beta_{1}},\;\ldots ,\text{ c}\hat{\beta_{\text{N}}}\right]}^{\text{t}}$, where *c* represents a contrast vector. Ignoring the subscript *v* for voxels, we estimate the following model:

(1) $$\begin{array}{r} {\text{Y}}_{\text{G}}\;=\;{\text{X}}_{\text{G}}\beta_{\text{G}}+\;\varepsilon_{\text{G}} \end{array}$$

in which X_G_ is a group design matrix and ε_G_ a mixed-effects zero mean error component containing between subject variability and within subject variability. In the simplest case, we are interested in the average group activation. Therefore, when testing the null hypothesis *H*_0_ of no group activation (β_G_ = 0), X_G_ is a column vector of length *N* with all elements equal to 1 and the test statistic is identical to a one-sample *t*-test:

(2) $$\text{T}\;=\;\;\frac{\hat{\beta_{\text{G}}}}{\sqrt{\hat{\text{var}(\hat{\beta_{\text{G}})}}}}$$

Under the assumption that $\varepsilon_{\text{G}}\;\sim \text{ N}\left(0,\;\sigma_{\text{G}}^{2}I\right)$, this test statistic follows a *t*-distribution under H_0_. Alternatively, it is possible to test differences between groups of subjects (e.g., patients vs. controls) by incorporating additional regressors in the group design matrix. As statistical tests are performed in all voxels simultaneously, adjustments for multiple testing need to be imposed.

Several methods are available to estimate β_G_ and var(β_G_) in model (2). We consider the Ordinary Least Squares (OLS), Fixed Effects (FE) and Mixed Effects (ME) approaches. In this study, we use the FSL (RRID:SCR_002823) software library (Smith et al., 2004) and therefore only outline the implementation of these methods as described in Woolrich et al. (2004). For a discussion of different implementations in other software packages, see Mumford and Nichols (2006).

### Ordinary least squares

In the OLS procedure (Holmes and Friston, 1998), one assumes that within subject variability is equal across all subjects (resulting in homogeneous residual variance). In the simple case of seeking group average activation, and as shown in Mumford and Nichols (2009), β_*G*_ in model (2) can be estimated as ${\hat{\beta }}_{\text{OLS}}\;=\;{\text{X}}_{\text{G}}^{-}{\text{Y}}_{\text{G}}$ where—denotes the pseudo inverse. The residual error variance $\sigma_{\text{OLS}}^{2}$ is estimated as ${\left({\text{Y}}_{\text{G}}-{\text{X}}_{\text{G}}{\hat{\beta }}_{\text{OLS}}\right)}^{\text{t}}\left({\text{Y}}_{\text{G}}-{\text{X}}_{\text{G}}{\hat{\beta }}_{\text{OLS}}\right)/\left(\text{N}-1\right)$, and therefore var(β_OLS_) can be estimated as ${\left({\text{X}}^{\text{t}}\text{X}\right)}^{-1}{\hat{\sigma }}_{\text{OLS}}^{2}$. Under the assumption of Gaussian distributed error terms, the resulting test is equal to a one-sample *t*-test with N−1 degrees of freedom (dof) on the contrast of parameter estimates Y_G_ obtained at the first level.

In FSL, this model is termed *mixed effects: simple OLS*.

### Fixed and mixed effects

Both for the fixed and mixed effects models, β_*G*_ in model (2) and *var*(β_*G*_) are estimated as follows:

(3) $$\begin{array}{r} \hat{{\text{β}}_{\text{G}}}\;=\;{\left({\text{X}}_{\text{G}}^{\text{t}}{\hat{\text{W}}}^{-1}{\text{X}}_{\text{G}}\right)}^{-1}{\text{X}}_{\text{G}}^{\text{t}}{\hat{\text{W}}}^{-1}{\text{Y}}_{\text{G}} \end{array}$$

(4) $$\begin{array}{r} \text{var}\left(\hat{{\text{β}}_{\text{G}}}\right)\;=\;{\left({\text{X}}_{\text{G}}^{\text{t}}{\hat{\text{W}}}^{-1}{\text{X}}_{\text{G}}\right)}^{-1} \end{array}$$

with W a weighting matrix. As is the case for OLS, the error terms in model 2 are typically assumed to follow a Gaussian distribution. In the fixed effects model, the weights in W correspond to the within subject variability only (ignoring between subject variability). Hence, W is an *N* × *N* matrix equal to:

(5) $$\begin{array}{r} \hat{\text{W}}\;=\;\left(\begin{array}{ccc} {\hat{\sigma }}_{1}^{2} & 0 & 0\\ 0 & \ddots & 0\\ 0 & 0 & {\hat{\sigma }}_{\text{N}}^{2} \end{array}\right) \end{array}$$

Thus, β_G_ is equal to a weighted average of the first level contrast parameters with the weights corresponding to the inverse of the within subject variances. These variances are easily estimated at the first level of the GLM procedure. The number of degrees of freedom in the fixed effects model equals the number of scans per subject times the sample size at the second level minus the number of estimated parameters. Note, FSL restricts the number of dof to a maximum of 1,000 and is set equal to 999 when no information on the number of scans at the first level is provided. In FSL, this model is termed *fixed effects*.

For the mixed effects model, between subject variability $\left(\sigma_{\eta }^{2}\right)$ is incorporated into the weighting matrix:

(6) $$\hat{\text{W}}\;=\;\left[\begin{array}{ccc} \left({\hat{\sigma }}_{\text{1}}^{\text{2}}+{\hat{\sigma }}_{\eta }^{\text{2}}\right) & 0 & 0\\ 0 & \ddots & 0\\ 0 & 0 & \left({\hat{\sigma }}_{\text{N}}^{\text{2}}+{\hat{\sigma }}_{\eta }^{\text{2}}\right) \end{array}\right]$$

Estimating the variance components of the mixed effects model is complicated as (1) multiple components need to be estimated and (2) there are typically only a few measurements on the second level to estimate $\sigma_{\eta }^{2}$. FSL relies on a fully Bayesian framework with reference priors (Woolrich et al., 2004). Inference on β_G_ in model (2) then depends on its posterior distribution, conditional on the observed data (Mumford and Nichols, 2006). As suggested in Woolrich et al. (2004), a fast approximation is used first and then on voxels close to significance thresholding a slower Markov-Chain-Monte-Carlo sampling framework is applied to estimate all parameters of interest. The posterior marginal distribution of β_G_ is assumed to approximate a multivariate *t*-distribution with non-centrality parameter $\hat{\beta_{\text{G}}}$. A lower bound on the number of degrees of freedom (i.e., *N*−*p*_G_ with *p*_G_ the amount of parameters in the group design matrix X_G_) is used for the voxels with a test statistic close to zero and an EM algorithm (Dempster et al., 1977) is employed to estimate the effective degrees of freedom in voxels that are close to the significance threshold. In FSL, this model is termed *mixed effects: FLAME1*+*2*.

### Coordinate-based meta-analyses

#### ALE

Coordinate based meta-analyses combine coordinates from several studies to assess convergence of the location of brain activation. The ALE algorithm (Turkeltaub et al., 2002, 2012) starts by creating an activation probability map for each study in the meta-analysis. The location of each reported peak in a study is modeled using a Gaussian kernel to reflect the spatial uncertainty of the peak activation. Voxels where kernels overlap due to multiple nearby peaks take the maximum probability. Next an ALE map is calculated by taking the voxelwise union of the probabilities over all studies. If *p*_*vm*_ is the probability of a peak at voxel *v* (*v* = 1, …, *S*) in a study *m* (*m* = 1, …, *K*), then the union is defined as: $1-\;\prod_{m\;=\;1}^{K}\left(1-p_{vm}\right)$. A null distribution created with non-linear histogram integration is used for uncorrected voxel-level inference under the assumption of spatial independence (Eickhoff et al., 2012). Various corrections for multiple comparisons are available in ALE, but based on the large-scale simulation study in Eickhoff et al. (2016a), cluster-level family-wise error (cFWE) correction is preferred as it provides the highest power to detect a true underlying effect while being less susceptible to spurious activation in the meta-analysis. All ALE calculations were implemented using MATLAB scripts which corresponds to the ALE algorithm as described in Eickhoff et al. (2009, 2012, 2016a) and Turkeltaub et al. (2012) provided to us by Prof. dr. Simon Eickhoff (personal communication).

#### Random effects CBMA

An alternative approach is to use the associated *t*-values of reported peaks to estimate corresponding effect sizes, enabling a weighted average of these effect sizes. Depending on the weights, this results in a random or fixed effects meta-analysis model. To evaluate the performance of these effect size based methods, we use the seed based *d*-mapping algorithm (SBdM), as described in Radua et al. (2012). However, we have carefully replicated this algorithm in R (R Development Core Team, 2015) to efficiently develop a fixed effects meta-analysis implementation (see below). The interested reader can find this implementation in the GitHub repository mentioned above. As we cannot exclude slightly divergent results compared to the standalone version of SBdM^4^, we choose to refer to this implementation as random effects CBMA. We follow the guidelines for significance testing as described in Radua et al. (2012). Unlike ALE, the method assigns effect sizes to voxels. These correspond to the standardized mean (for a one sample design) known as Hedges' *g* (Hedges, 1981) obtained from the peak height *t*_*vm*_ in study *m* (*m* = 1, …, *K*) and voxel *v* (*v* = 1, …, *S*). For a given peak with height *t*_*vm*_ stemming from a one-sample *t*-test and *N*_*m*_ subjects, the effect size *g*_*vm*_ and a correction factor *J*_*m*_ is given by:

(7) $$\begin{array}{r} g_{vm}\;=\;\frac{t_{vm}}{\sqrt{N_{m}}}\;\times \;J_{m} \end{array}$$

(8) $$\begin{array}{r} J_{m}\;=\;1-\left(\frac{3}{\left(4\;\times \left(N_{m}-1\right)\right)-1}\right) \end{array}$$

First, all coordinates of local maxima are smoothed using an unnormalized Gaussian kernel. The resulting map represents for each voxel the distance to a nearby peak. Effect sizes in voxels surrounding a peak are then obtained through multiplication of the peak effect size calculated using Equation (7) and the smoothed map. The effect size in voxels where kernels overlap is an average weighted by the square of the distance to each nearby peak. Once an effect size $g_{vm}^{*}$ (i.e., the smoothed standardized effect size) is obtained in each voxel (which will be zero for voxels that are not near a peak), the variance of this effect size is obtained as follows (Hedges and Olkin, 1985):

(9) $$\begin{array}{r} \text{var}\left(g_{vm}^{*}\right)\;=\;\frac{1}{N_{m}}+\left[1-\;{\left(\frac{\Gamma \left(\frac{\left(N_{m}-2\right)}{2}\right)}{\Gamma \left(\frac{\left(N_{m}-1\right)}{2}\right)}\right)}^{2}\;\times \;\frac{\left(N_{m}-3\right)}{2}\right]\times g^{*2} \end{array}$$

Combining all studies proceeds by calculating the weighted average θ through a random effects model:

(10) $$\begin{array}{r} \theta_{v}\;=\;\frac{\sum_{m\;=\;1}^{K}U_{vm}\times g_{vm}^{*}}{\sum_{m\;=\;1}^{K}U_{vm}} \end{array}$$

with the weights in *U*_*vm*_ being the inverse of the sum of both the within study variability (estimated using Equation 9) and the between study variability (τ^2^). The latter is estimated through the DerSimonian and Laird estimator (DerSimonian and Laird, 1986).

In a final step, the null hypothesis *H*_0_:θ_*v*_ = 0 is calculated with the following *Z*-test: $Z_{v}\;=\;\theta_{v}/\sqrt{1/\left(\overset{K}{\underset{m\;=\;1}{\sum }}U_{vm}\right)}$ (Borenstein et al., 2009). A permutation approach with 20 iterations is used to create a combined null-distribution, in which each iteration is a whole brain permutation with close to 100,000 values. To optimally balance sensitivity and specificity, a threshold of *P* = 0.005 and *Z* > 1 is recommended, instead of classical multiple comparisons corrections (Radua et al., 2012). Since the effect size is imputed as 0 in voxels far from any peak, *Z* > 1 is a lot more unlikely under the empirical null distribution.

#### Fixed effects CBMA

**Figure 1:**
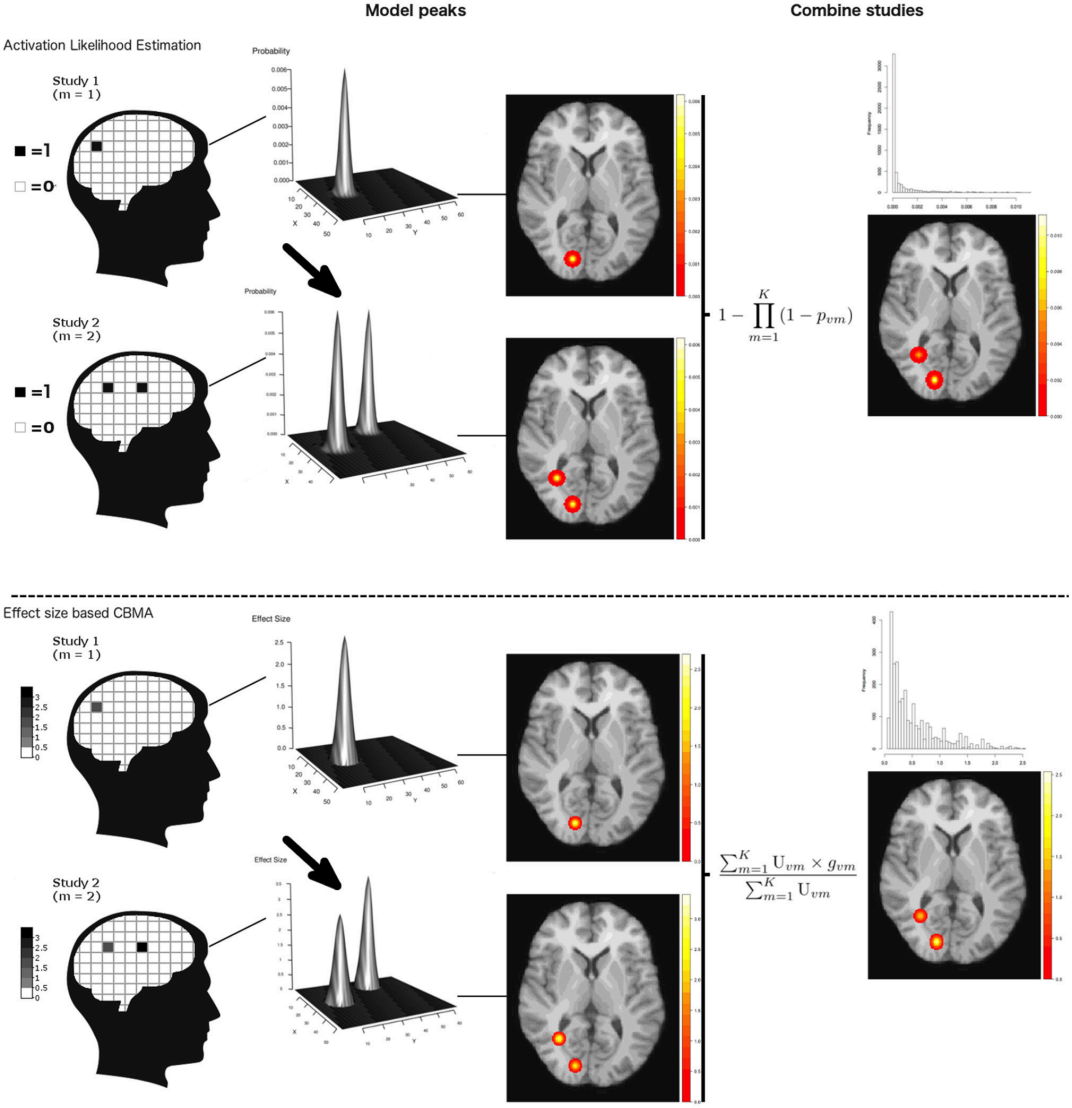
Illustration of ALE and an effect size based CBMA. Reported coordinates are first modeled by applying a Gaussian kernel. These are then combined either through calculating probabilities or by transforming the test-statistics to effect sizes and calculate a weighted average. Note that for illustration purpose, we only plot the values > 0 in the histograms. Illustration is prior to thresholding.

Finally, we also evaluate the performance of a fixed effects CBMA. This procedure only differs from the random effects CBMA with respect to the weights. A fixed effects model ignores heterogeneity across studies and only uses the within study variability to calculate the weights, *U*_*vm*_. An illustration of ALE and an effect size based CBMA prior to thresholding can be seen in Figure 1.

### Design

In this section, we describe the set-up of our study to test the effect of pooling subjects at the individual study level on the outcome of methods for CBMA.

#### Resampling scheme

**Figure 2:**
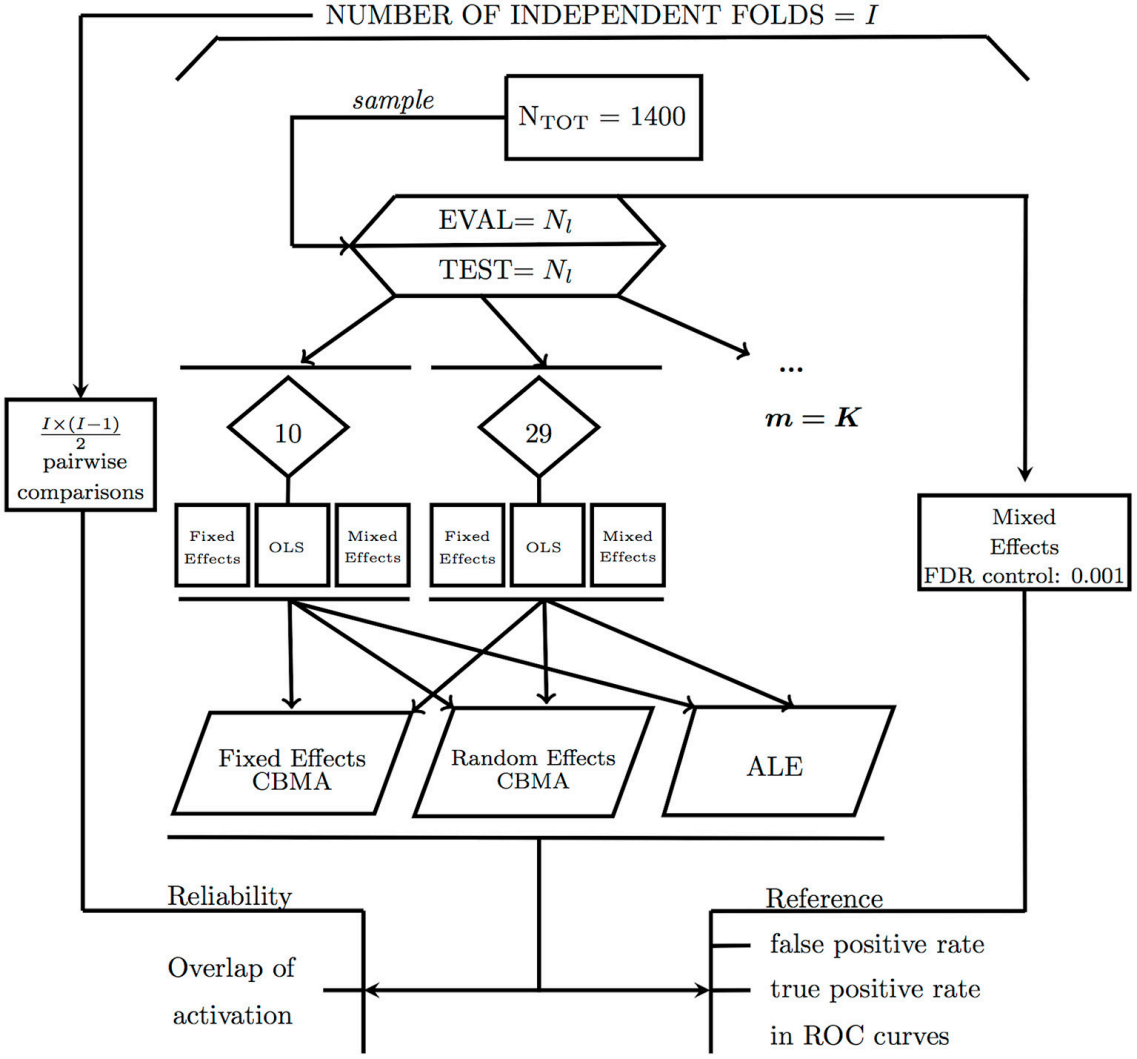
Design of the study illustrating the calculation of false positives and true positives and reliability using an evaluation condition (EVAL) and test condition (TEST).

The general study design is depicted in Figure 2. To assess the activation reliability of the outcome of the methods for CBMA, we need to start with creating independent subsets of subjects called folds. In one-fold *l* (*l* = 1, …, *I*), *N*_*l*_ subjects are sampled without replacement into an evaluation condition while *N*_*l*_ different subjects go into a test condition. Next, the subjects in the test condition are subsampled into *K* smaller studies with varying sample sizes (mean = 20, *SD* = 5). No subsampling restriction into the *K* studies is imposed. Each fold is used once as a test condition and once as an evaluation condition. No fold can simultaneously be the test and evaluation condition.

Note that our design results in a trade-off between the number of independent folds (*I*) and the number of subjects per fold (*N*_*l*_). Moreover, we also vary the number of studies in the meta-analysis. In Table 1, we list the several scenarios of the resampling scheme. It contains the number of studies (*K*), the corresponding number of maximum independent folds (*I*) and the corresponding sample size (*N*_*l*_). Note that the maximum amount of studies equals *K* = 35 as we then use all subjects in the database to construct an independent test and evaluation condition.

**Table 1:** Overview of the different designs considered.

**Studies (*K*)**	**Folds (*I*)**	**Sample size (N_l_)**
10	7	200
12	5	240
14	5	280
16	4	320
18	3	360
20	3	400
30	2	600
35	2	700

*The number of studies in the meta-analysis determines the number of independent folds and subsequent total sample size in the test and evaluation condition*.

#### Test condition

The *K* studies in the test condition are all analyzed using FSL, version 5.0.6. Every second level GLM model (FE, OLS, and ME) is fitted to each of the *K* studies with the FLAME 1 + 2 option for the mixed effects models. We only test for average group activation.

To obtain local maxima, we search for clusters of significant activity in the *K* studies of the test condition because clusters provide an intuitive way of defining local maxima (i.e., the highest peak within each cluster). To control for multiple testing, we first determine a threshold such that the voxelwise false discovery rate (FDR) is controlled at level 0.05. Then, we determine clusters using a 26-voxel neighborhood algorithm. By doing so, we obtain local maxima, but avoid clusterwise inference which is shown to be conservative (Eklund et al., 2016) for event-related designs and FSL's mixed effects group level models. The average observed FDR threshold in this study equals *Z* = 3.18. The resulting coordinates of the foci from each study with the number of subjects are then used as input for the ALE meta-analysis. The corresponding *t*-values (peak heights) are added for the fixed and random effects coordinate-based meta-analyses. To identify significant voxels in the resulting meta-analyses, we apply the recommended procedures as described in section Coordinate-Based Meta-Analyses. For ALE, a voxelwise threshold uncorrected for multiple testing is used at level 0.001, as well as a cluster-level family-wise error (cFWE) correction for multiple testing at level 0.05. For the fixed and random effects CBMA we use a threshold at *Z*>1 and at *P* = 0.005, uncorrected for multiple testing. To complement the comparison between the different methods for CBMA, we include an analysis with fixed and random effects CBMA with a threshold at *P* = 0.001. Since this thresholding procedure for the fixed and random effects CBMA has not yet been validated (Radua et al., 2012), we refer to this analysis in the Supplementary Material, section 6.

#### Evaluation condition

Finally, the *N*_*l*_ subjects in the evaluation condition are combined in one large, high powered study, using a mixed effects model. To control for multiple testing and balance sensitivity and specificity in this large sample, we apply a more conservative threshold such that the voxelwise FDR is controlled at level 0.001. The resulting map serves as a reference/benchmark image for the meta-analysis results obtained in the test condition. Note that a threshold for the sample in the evaluation condition could be chosen in different ways so deviations from the benchmark image should not be interpreted in an absolute manner but compared between methods in a relative manner. To this extent, we also compare the test condition with two evaluation conditions using different thresholds. One using a voxelwise FDR at level 0.05 and a second using an uncorrected threshold at level 0.001. For sparseness, we include these results in the Supplementary Material. Furthermore, we do not model all available subjects into the evaluation condition, but a set of *N*_*l*_ different subjects with respect to the test condition. This ensures that the evaluation condition is based on independent data. Next, by having an equal sample size in both conditions one can consider the evaluation condition as a perfect scenario in which all data is available for aggregation, while the test condition is the scenario in which we need to aggregate censored summary results in the form of peak coordinates.

### Performance measures

To assess the performance of the different procedures for CBMA, we use two different measures: the balance between false positives and true positives in receiver operator characteristic (ROC) curves and activation reliability as a proxy for replicability.

#### ROC curves

Statistical tests are often evaluated based on the extent to which they are able to minimize the number of false positives (detecting signal where there is none) while maximizing the amount of true positive hits (detecting true signal). Receiver operator characteristic (ROC) curves plot the observed true positive rate (TPR) against the observed false positive rate (FPR) as the threshold for significance (α) is gradually incremented. To calculate true and false positives, we compare the results from the meta-analysis in the test condition with the reference image in the evaluation condition (EVAL on Figure 2). The TPR or sensitivity is calculated as the number of voxels that are statistically significant in both the meta-analysis map and the reference map divided by the total number of voxels that is statistically significant in the reference map. The FPR or fall-out is calculated as the number of voxels that is statistically significant in the meta-analysis map but not in the reference map divided by the total number of voxels that is NOT statistically significant in the reference map.

Because the TPR and FPR are calculated voxelwise, we construct the ROC curves based on uncorrected *p*-values for the meta-analyses by incrementing the significance level, alpha, from 0 to 1. Finally, we average over the *I* folds the individual ROC curves and additionally use the area under the curve (AUC) as a summary measure. Higher AUC values indicate a better balance in discriminating between false positive and true positive voxels.

Since the ALE algorithm uses an MNI brain template with a higher resolution (2 mm voxels, dimensions 91 × 109 × 91) than the (pre-processed) IMAGEN data (3 mm voxels, dimensions 53 × 63 × 46), the reference image is also resampled to a higher resolution so that it matches the resolution of the ALE images. We apply a linear affine transformation with 12 degrees of freedom from the EPI template of the IMAGEN dataset to the MNI brain template, using a correlation ratio cost function (Jenkinson et al., 2002) and trilinear interpolation in FSL. As the fixed and random effects meta-analyses model the local maxima using the same brain template as the IMAGEN data, no such transformation is needed here to calculate the ROC curves.

#### Reliability

We consider activation reliability as an indicator for the success of replicating results. We define replicability as the ability to repeat the results of an experiment using the exact same materials, procedures and methods, but with a different set of subjects. There is no consensus in the literature on this definition as other authors use terms such as strong replicable results or direct reproduction to indicate the same concept (Pernet and Poline, 2015; Patil et al., 2016). We quantify reliability in two ways. First, we measure the overlap of results between folds. We calculate the percent overlap of activation (Maitra, 2010) between all $\frac{I\;\times \;\left(I\;-\;1\right)\;}{2}\;$ pairwise combinations of the *I* unique folds of the design (Figure 2). Let *V*_*a,b*_ represent the intersection of statistically significant voxels in image *a* and *b*, *V*_*a*_ the amount of statistically significant voxels in image *a* and *V*_*b*_ the amount of statistically significant voxels in image *b*. The overlap ω_*a,b*_ is then defined as:

(11) $$\begin{array}{r} \omega_{a,b}\;=\;\frac{V_{a,b}}{V_{a}+V_{b}-\;V_{a,b}} \end{array}$$

This measure ranges from 0 (no overlap) to 1 (perfect overlap). Note that this is an adaptation of the Dice (1945) or the Sørensen (1948) similarity coefficient.

As a second method to quantify reliability, we describe the amount of unique information captured in each fold. We first quantify the number of times out of the *I* folds a voxel is declared significant and visualize this on a heatmap. We do the same for the *I* reference images from the evaluation condition. As a comparison, we include the average effect size map obtained using again the reference images. Next, we run a 26-point search neighboring algorithm on each thresholded meta-analysis to calculate the frequency of clusters of at least one statistically significant voxel. We record the average cluster size expressed in number of voxels. We then assess the number of unique clusters across the pairwise combinations. A cluster of statistically significant voxels in image *a* is unique if no single voxel from this cluster overlaps with a cluster of statistically significant voxels in the paired image *b*. We finally determine the amount of these unique clusters that are large (we have set the threshold for large at 50 voxels) and divide this by the total amount of statistically significant clusters to obtain the proportion of large unique clusters. Additionally, we study the number of clusters and cluster sizes for both unique and overlapping clusters to get an overview, independent of the chosen threshold on the cluster size. Given a sample size, smaller amounts of (large) unique clusters imply a higher pairwise reliability. For sparseness, we limit these calculations to *K* = 10, 20, and 35.

## Results

### ROC curves

**Figure 3:**
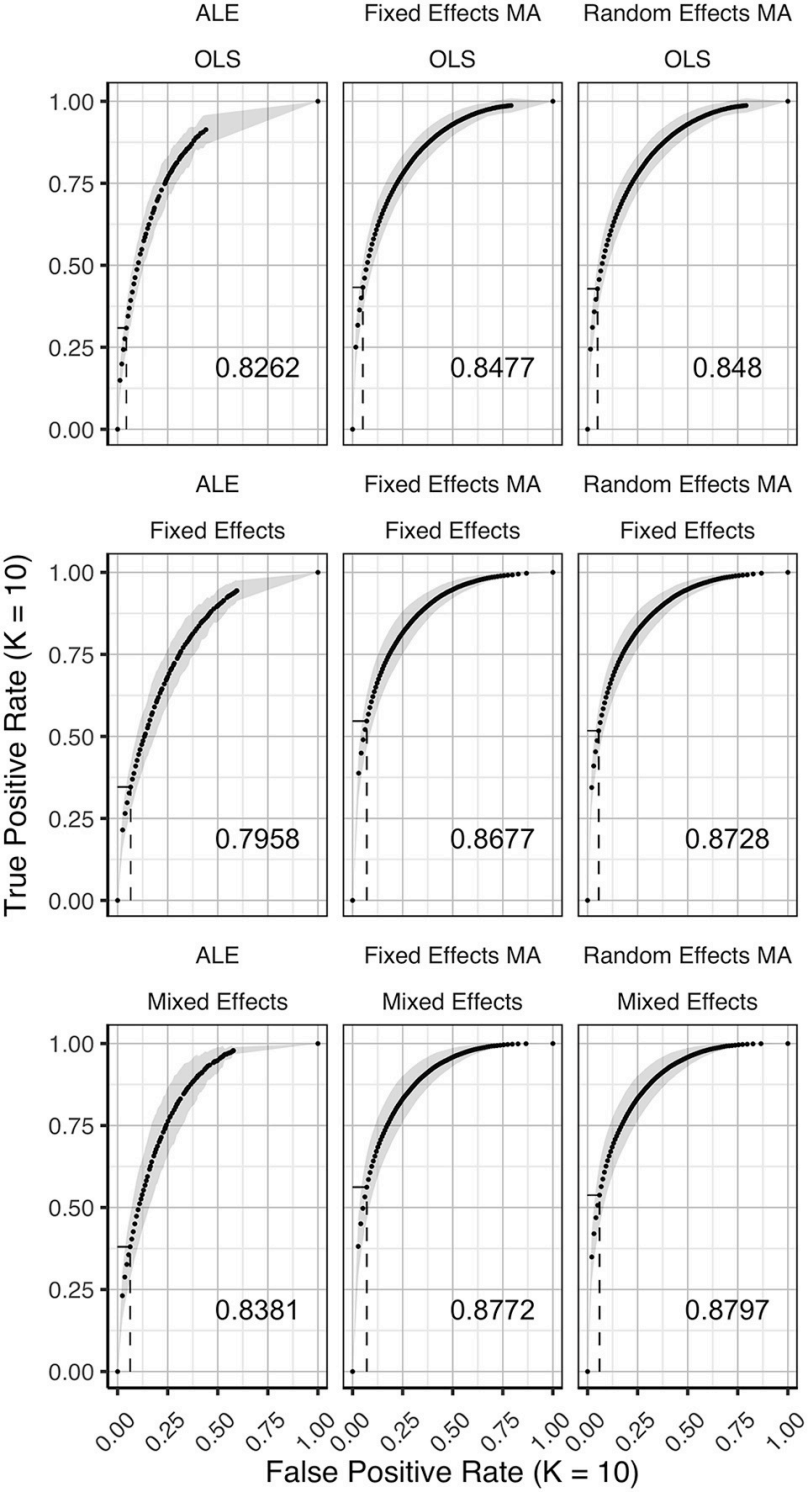
ROC curves (±1 standard deviation), averaged over *I* = 7 folds plotting the observed true positive rate against the observed false positive rate for *K* = 10. The columns correspond to the coordinate-based meta-analyses (left, ALE uncorrected procedure; middle, fixed effects meta-analysis; right, random effects meta-analysis). The rows correspond to the second level GLM pooling models (**top**, OLS; **middle**, fixed effects; **bottom**, mixed effects). For each of those, the area under the curve (AUC) is calculated and shown within the plot. The drop-down lines correspond to the point at which the pre-specified nominal level is set at an uncorrected α level of 0.05.

**Figure 4:**
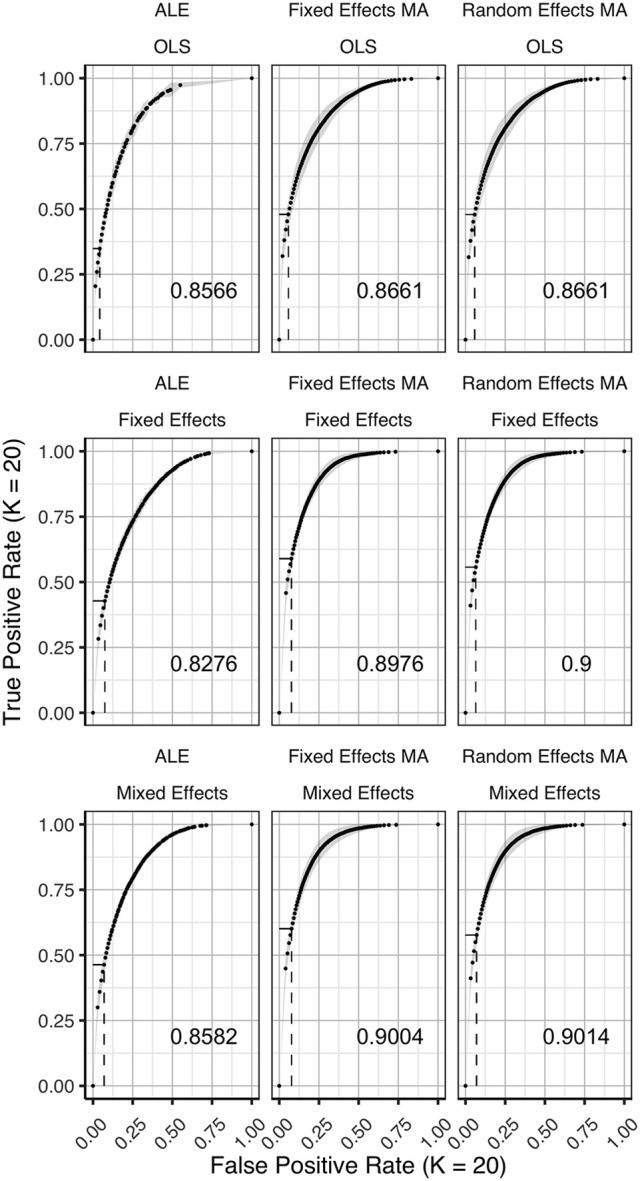
ROC curves (±1 standard deviation), averaged over I = 3 folds plotting the observed true positive rate against the observed false positive rate for *K* = 20. The columns correspond to the coordinate-based meta-analyses (left, ALE uncorrected procedure; middle, fixed effects meta-analysis; right, random effects meta-analysis). The rows correspond to the second level GLM pooling models (**top**, OLS; **middle**, fixed effects; **bottom**, mixed effects). For each of those, the area under the curve (AUC) is calculated and shown within the plot. The drop-down lines correspond to the point at which the pre-specified nominal level is set at an uncorrected α level of 0.05.

**Figure 5:**
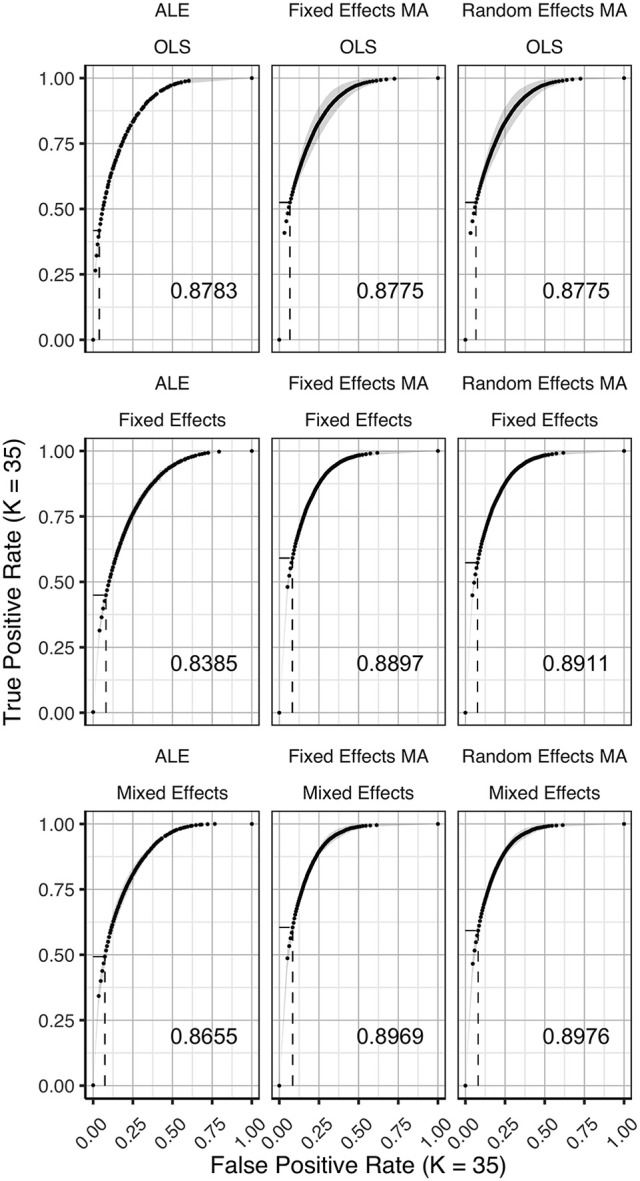
ROC curves (±1 standard deviation), averaged over *I* = 2 folds plotting the observed true positive rate against the observed false positive rate for *K* = 35. The columns correspond to the coordinate-based meta-analyses (left, ALE uncorrected procedure; middle, fixed effects meta-analysis; right, random effects meta-analysis). The rows correspond to the second level GLM pooling models (**top**, OLS; **middle**, fixed effects; **bottom**, mixed effects). For each of those, the area under the curve (AUC) is calculated and shown within the plot. For each of those, the area under the curve (AUC) is calculated and shown within the plot. The drop-down lines correspond to the point at which the pre-specified nominal level is set at an uncorrected α level of 0.05.

**Figure 6:**
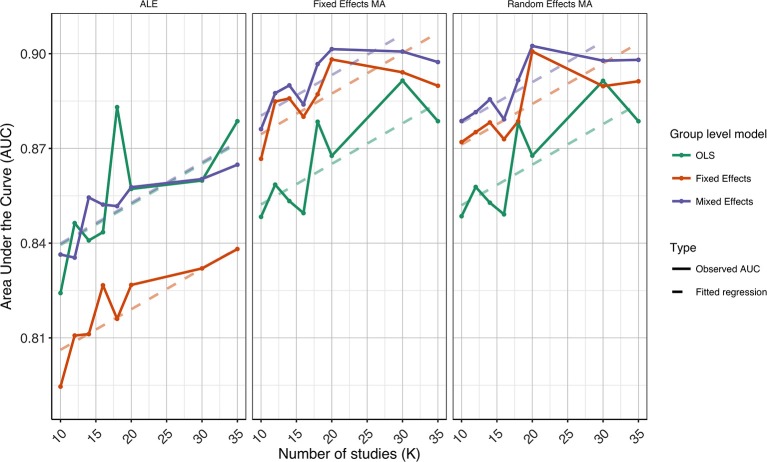
Values of the area under the curve averaged over all folds for all *K*. The 3 panes correspond to the methods of CBMA while the 3 colors correspond to the group models. Solid lines are the observed values of the AUC. The dashed lines correspond to the fitted regression lines.

In Figures 3–5 we present the average ROC curves (over folds) that show the observed true positive rate against the observed false positive rate for selected values of *K* = 10, 20, and 35 over the entire range of α. ROC curves for all values of *K* are included in the Supplementary Material, section 1. Some readers might prefer to look at ROC curves for which α ∈[0, 0.1] and the standardized partial AUC (McClish, 1989). We include these figures in the Supplementary Material, section 2. We observe the same patterns when ∈[0, 0.1]. Recall that given comparisons are made with the reference image, all values should be used for relative comparisons as the absolute AUC will depend on how the reference image is determined. We refer to section 3 and 4 in the Supplementary Material for ROC curves based on reference images with different levels of statistical thresholding. In general, these curves show similar relative results as those presented below. Finally, we plot the average AUC for each *K* in Figure 6.

To formally test the effect of the group level models, the methods for CBMA and *K* on the AUC, we also fit a linear mixed model with a random intercept for group level models. We use Wald statistical tests for the main effects and the interaction effect between group level models and the methods for CBMA. Results reveal significant main effects for the group level model ($\chi_{2}^{2}\;=\;28.68$, *P* < 0.001), the methods for CBMA ($\chi_{2}^{2}\;=\;155.00$, *P* < 0.001) and *K* ($\chi_{1}^{2}\;=\;36.01$, *P* < 0.001). Furthermore, we observe a significant interaction effect between the group level models and the models for meta-analysis ($\chi_{4}^{2}\;=\;48.10$, *P* < 0.001). No other two-way or three-way interaction effects are significant. These terms are subsequently excluded from the model. The fitted regression lines are also shown in Figure 6.

We observe higher values for the AUC using fixed and random effects models compared to ALE. The only exception is observed for the combination of OLS and ALE for *K* = 18 and 35. Only small differences are observed between the fixed and random effects meta-analysis. The observed TPR at an uncorrected threshold of 0.05 never exceeds 0.5 for ALE in any of the scenarios, while the TPR of the fixed and random effects CBMA methods approaches 0.6 when combining mixed or fixed effects group level models with a higher amount of studies in the meta-analysis.

As can be seen in Figure 6, the OLS model (compared to fixed and mixed effects group models) is associated with lower values of the AUC in the fixed and random effects meta-analysis. Using ALE on the other hand, we observe consistently low values using fixed effects group level models. The mixed effects group models outperform the fixed effects models. For two cases (*K* = 18 and 35, ALE) does an OLS group model outperform the mixed effects model.

Finally, for all CBMA methods, increasing the number of studies in the meta-analysis results in a higher AUC. The average AUC of the meta-analyses, regardless of the group level models, increases for *K* = 10 from 0.82 (ALE), 0.86 (fixed effects MA) and 0.87 (random effects MA) to respectively 0.85, 0.89, and 0.89 in *K* = 20. Adding even more studies (*K* = 35) is associated with a further increase to 0.86 of the average AUC for ALE, but not for the fixed (0.89) and random effects (0.89) meta-analyses. Also note the ceiling effect when *K* ≥ 20 for the fixed and random effects meta-analyses using mixed effects group models.

Overall, the best balance between TPR and FPR detection is observed when using mixed effects group level models together with a fixed or random effects meta-analysis.

### Reliability

**Figure 7:**
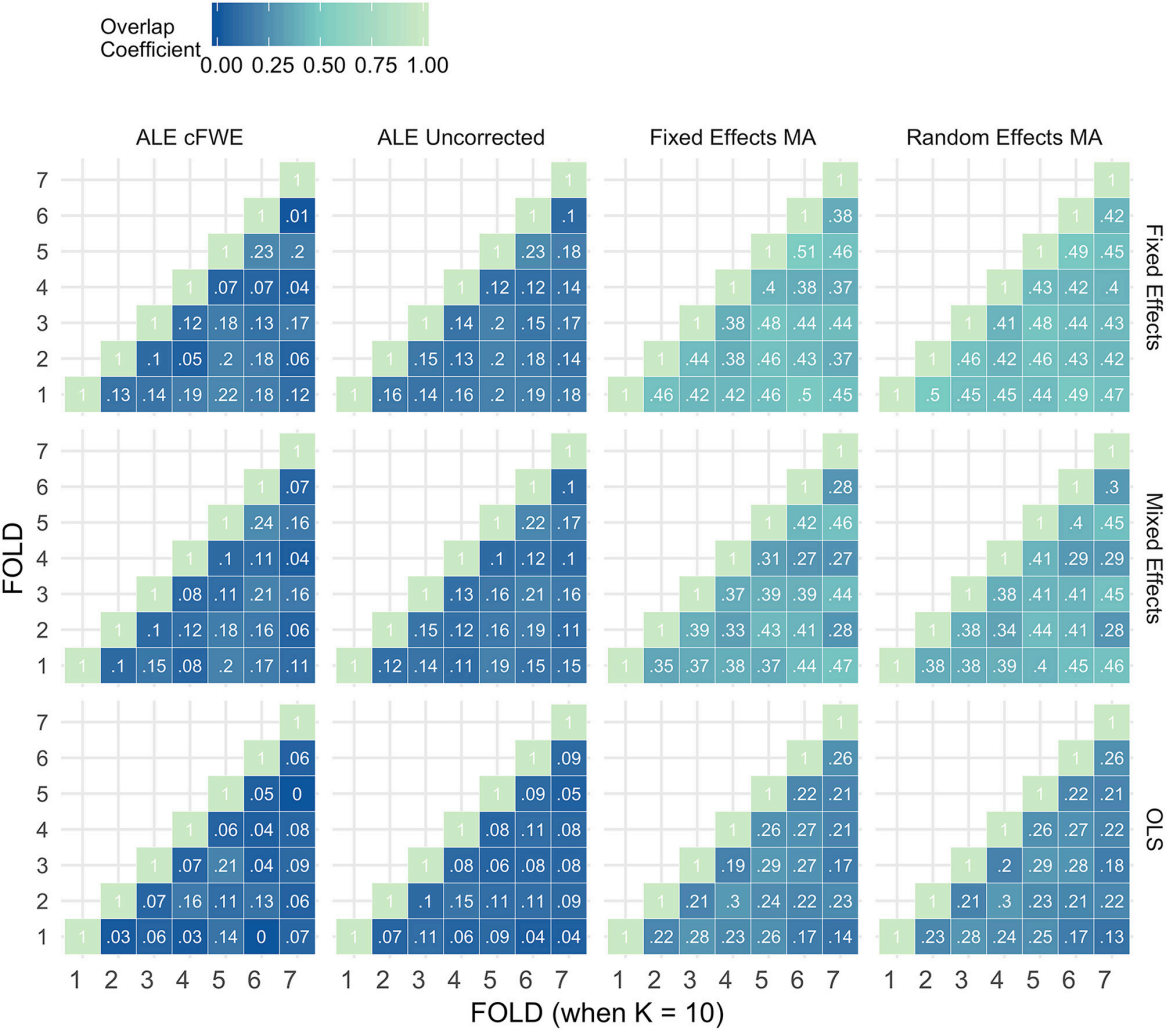
Percent overlap of activation (ω_*a,b*_) from all pairwise comparisons for *K* = 10. The rows represent the group level models (top to bottom: fixed effects, mixed effects and OLS). The columns represent the thresholded meta-analyses. From left to right: ALE cFWE at 0.05, ALE uncorrected at 0.001 and fixed and random effects CBMA at 0.005 with *Z* > 1.

**Figure 8:**
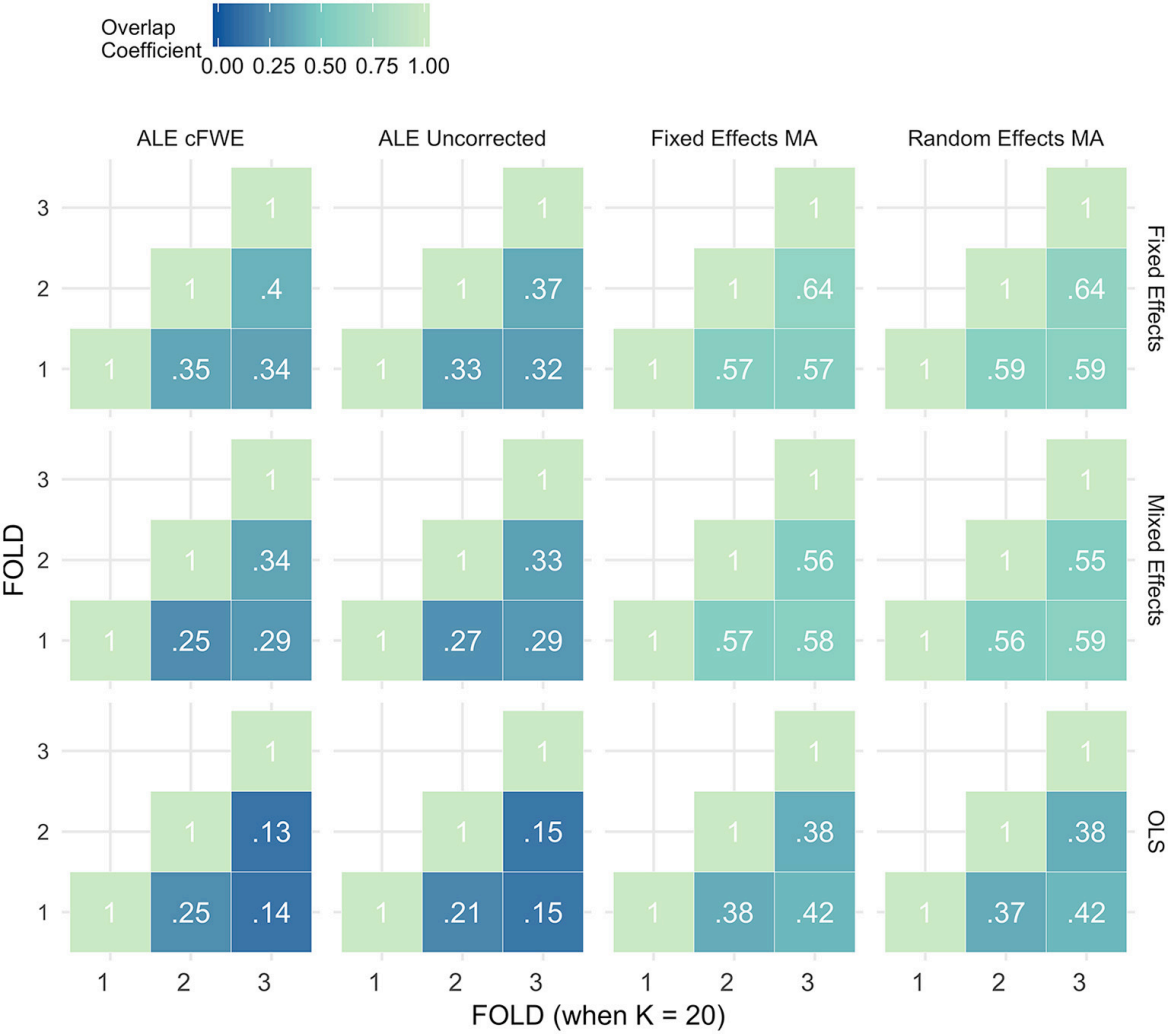
Percent overlap of activation (ω_*a,b*_) from all pairwise comparisons for *K* = 20. The rows represent the group level models (**top** to **bottom**: fixed effects, mixed effects and OLS). The columns represent the thresholded meta-analyses. From left to right: ALE cFWE at 0.05, ALE uncorrected at 0.001 and fixed and random effects CBMA at 0.005 with *Z* > 1.

**Figure 9:**
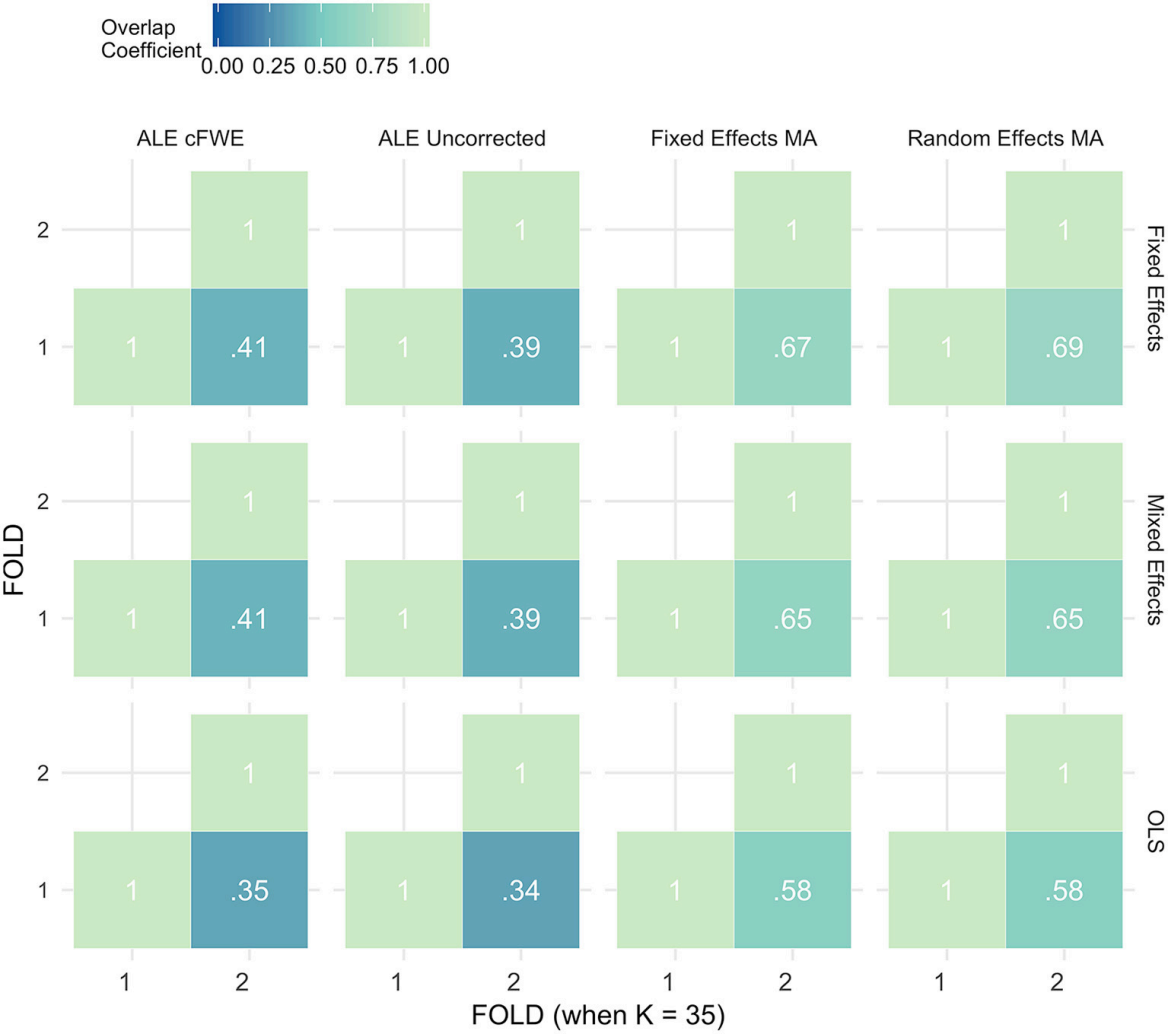
Percent overlap of activation (ω_*a,b*_) from all pairwise comparisons for *K* = 35. The rows represent the group level models (**top** to **bottom**: fixed effects, mixed effects and OLS). The columns represent the thresholded meta-analyses. From left to right: ALE cFWE at 0.05, ALE uncorrected at 0.001 and fixed and random effects CBMA at 0.005 with *Z* > 1.

**Figure 10:**
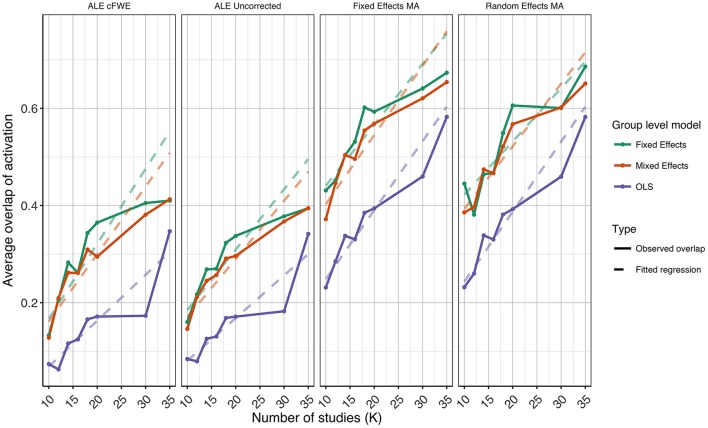
Values of the percent overlap of activation, averaged over all folds for all *K*. The 4 panes correspond to the methods of CBMA (after significance testing) while the three colors correspond to the group models. Solid lines are the observed values of overlap. The dashed lines correspond to the fitted regression lines.

Figures 7–9 display the percent overlap of activation for *K* = 10, 20, and 35. We refer to the Supplementary Material, section 5 for other values of *K*. We plot the average overlap between independent folds for all values of *K* in Figure 10. Furthermore, we compare the thresholded outcome images of the methods for CBMA at *P* = 0.001. However, we do not observe differences from the results using the recommended procedures for statistical inference. Hence, the average overlap for all values of *K* using the (uncorrected) statistical threshold is given in the Supplementary Material, section 6.

Noticeably, the overlap values have a wide range from 0.07 (OLS, ALE cFWE, *K* = 10) to a moderate 0.69 (fixed effects group level model, random effects MA, *K* = 35). Average overlap values over *I* folds and the group level models/CBMA methods can be found in Table 2. Again, as the overlap between thresholded maps depends on the chosen threshold, it is better to focus on the relative performances of the group level models and methods for CBMA.

Table 2Averaged overlap values over the I folds and the CBMA methods (top) and over the I folds and the group level models (bottom) for each *K*.

**Table:** 

***K***	**Fixed effects**	**Mixed effects**	**OLS**
**AVERAGE OVERLAP OVER*I*AND CBMA METHODS**			
10	0.29	0.26	0.15
20	0.48	0.43	0.28
35	0.54	0.52	0.46

**Table:** 

***K***	**Fixed Effects MA**	**Random Effects MA**	**ALE Uncorrected**	**ALE cFWE**
**AVERAGE OVERLAP OVER*****I*****AND GROUP LEVEL MODELS**				
10	0.34	0.35	0.13	0.11
20	0.52	0.52	0.27	0.28
35	0.64	0.64	0.38	0.39

As with the AUC, we fit a linear mixed model with a random intercept for group level models on the measured overlap between folds. Results reveal significant main effects for the group level model ($\chi_{2}^{2}\;=\;1199.84$, *P* < 0.001), the methods for CBMA ($\chi_{3}^{2}\;=\;3547.68$, *P* < 0.001) and *K* ($\chi_{1}^{2}\;=\;1010.09$, *P* < 0.001). Furthermore, we observe a significant interaction effect between the group level models and the models for meta-analysis ($\chi_{6}^{2}\;=\;45.95$, *P* < 0.001) and a significant 3-way interaction effect between group level models, models for CBMA and *K* ($\chi_{6}^{2}\;=\;15.88$, *P* = 0.014). No other interaction terms are significant. The fitted regression line is plotted in Figure 10.

Similar to the ROC curves, we observe higher overlap when more studies are added to the meta-analysis. Furthermore, both ALE thresholding methods are associated with lower values of overlap compared to the fixed and random effects meta-analysis. In contrast to the ROC curves, the maximum overlap value observed in ALE is low and does not approach the performance of the fixed and random effects meta-analysis. We only observe small differences between the fixed and random effects meta-analysis. For *K* = 10, we observe mostly higher values using a random effects meta-analysis.

Regarding the group level models, OLS models are associated with lower coefficients of overlap than fixed and mixed effects models. In general, we observe higher values using fixed effects models compared to mixed effects models, though these differences are much smaller. These patterns are similar regardless of the CBMA method and study set size *K*.

**Figure 11:**
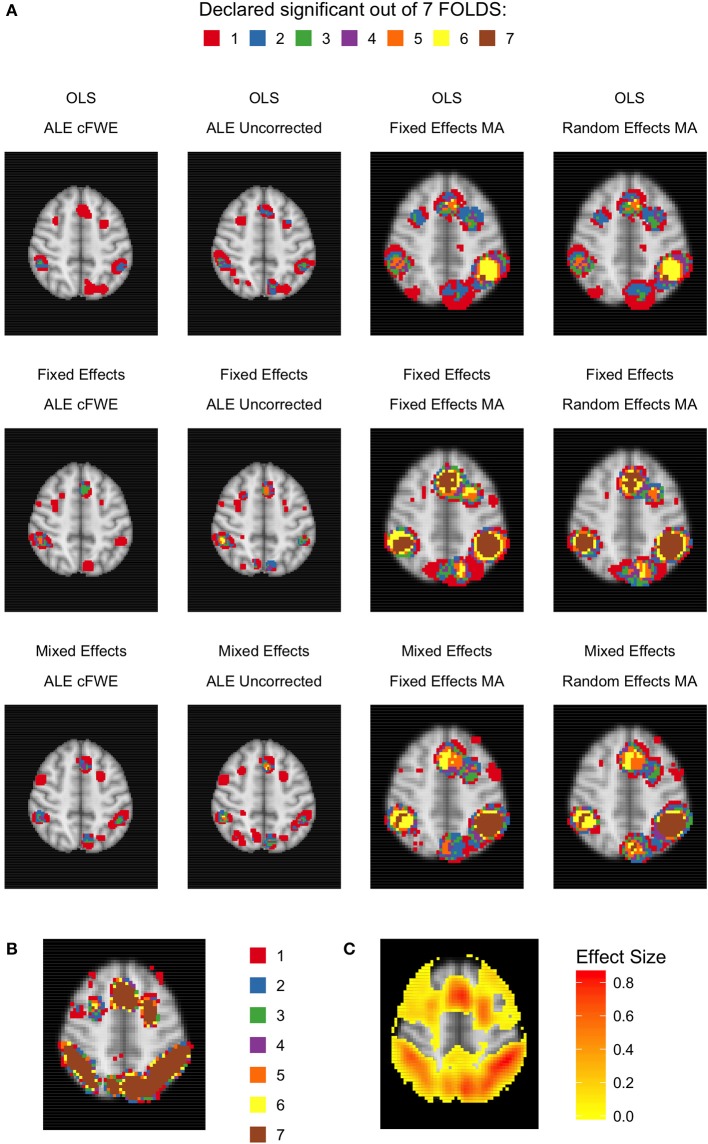
Heatmaps of MNI z-coordinate 50 for *K* = 10. **(A)** The number of folds in which each voxel has been declared statistically significant for each combination of a group level model (row-wise) and thresholded meta-analysis (column-wise). Note that the resolution of the images corresponding to the analyses within either ALE or the fixed and random effects CBMA is different (see main text). **(B)** The number of folds in which each voxel of the reference images has been declared statistically significant. Areas of interest involve the supramarginal gyrus (posterior division), superior parietal lobule and angular gyrus. **(C)** Average effect size of the reference images over the folds.

**Figure 12:**
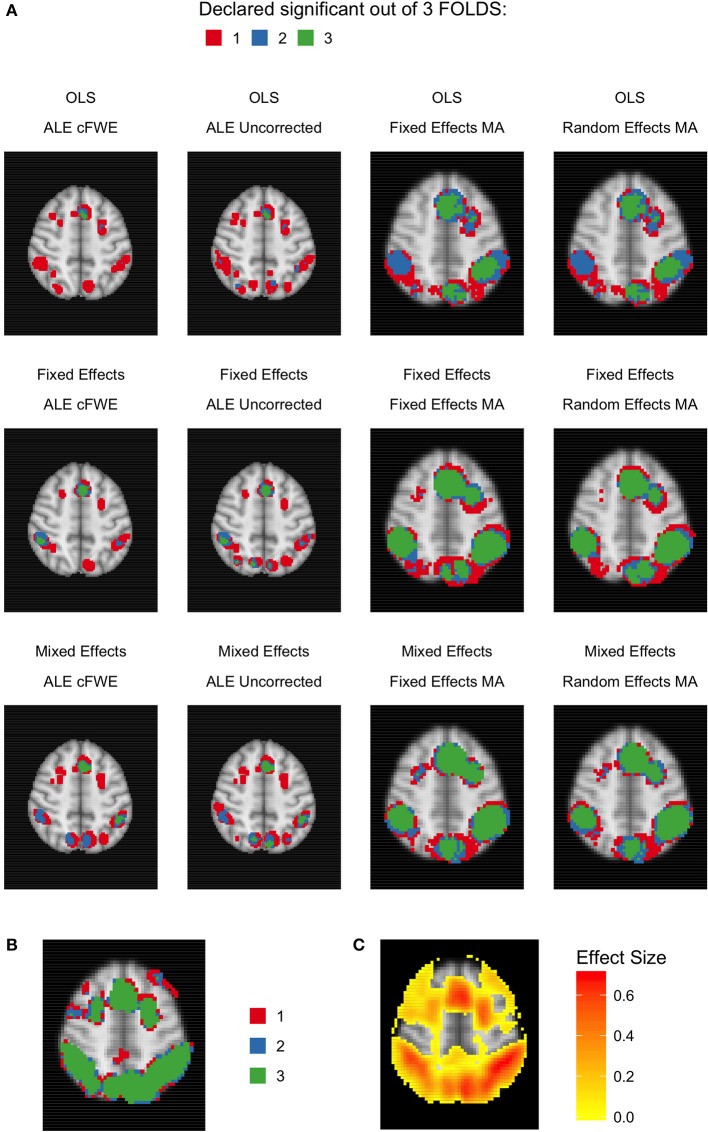
Heatmaps of MNI z-coordinate 50 for *K* = 20. **(A)** The number of folds in which each voxel has been declared statistically significant for each combination of a group level model (row-wise) and thresholded meta-analysis (column-wise). Note that the resolution of the images corresponding to the analyses within either ALE or the fixed and random effects CBMA is different (see main text). **(B)** The number of folds in which each voxel of the reference images has been declared statistically significant. Areas of interest involve the supramarginal gyrus (posterior division), superior parietal lobule and angular gyrus. **(C)** Average effect size of the reference images over the folds.

**Figure 13:**
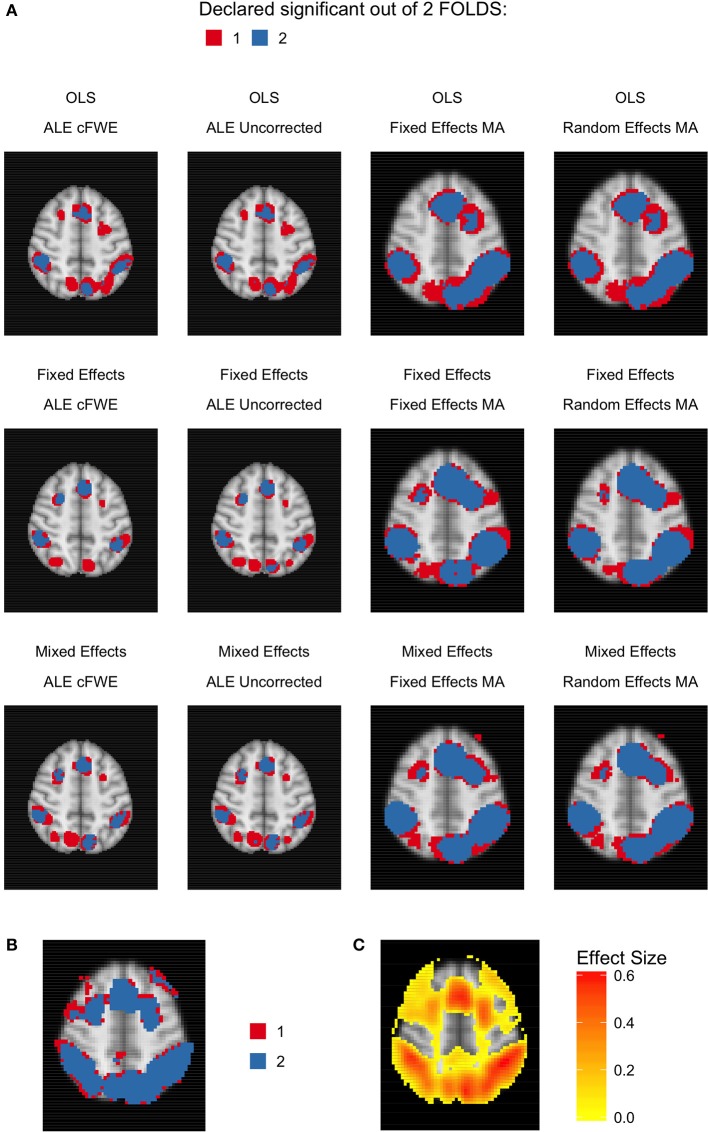
Heatmap of MNI z-coordinate 50 for *K* = 35. **(A)** The number of folds in which each voxel has been declared statistically significant for each combination of a group level model (row-wise) and thresholded meta-analysis (column-wise). Note that the resolution of the images corresponding to the analyses within either ALE or the fixed and random effects CBMA is different (see main text). **(B)** The number of folds in which each voxel of the reference images has been declared statistically significant. Areas of interest involve the supramarginal gyrus (posterior division), superior parietal lobule and angular gyrus. **(C)** Average effect size of the reference images over the folds.

Given the results on the overlap values, we look for similar patterns using the heatmaps at MNI *z*-coordinate 50 for *K* = 10 (Figure 11A), *K* = 20 (Figure 12A), and *K* = 35 (Figure 13A) and in the results detailing the amount of unique information in each iteration (Table 3).

**Table 3:** Descriptive results of the thresholded meta-analyses in a replication setting.

**K**	**Group Model**	**Meta-analysis**	***I***	**Amount of clusters**		**Voxels in clusters**		**Unique clusters**		**Large uni. clust**.		**Percentage**
				**Mean**	***sd***	**Mean**	***sd***	**Mean**	***sd***	**Mean**	***sd***	**Large clusters**
10	Fixed Effects	Fixed Effects MA	7	23.1	6.1	206.7	71.4	11.67	5.83	2.00	1.48	0.09
		Random Effects MA	7	19.6	3.1	186.4	43.9	9.33	2.88	1.45	1.52	0.08
		ALE Uncorrected	7	50.3	7.2	53.1	11.1	31.10	6.66	4.14	2.72	0.08
		ALE cFWE	7	11.0	1.3	155.0	31.8	5.71	1.94	5.71	1.94	0.52
	OLS	Fixed Effects MA	7	19.9	6.1	132.1	59.6	11.86	4.95	2.69	2.41	0.14
		Random Effects MA	7	20.6	7.4	126.6	68.6	12.43	5.92	2.62	2.23	0.13
		ALE Uncorrected	7	31.9	6.8	41.7	15.2	22.95	5.65	3.17	2.04	0.10
		ALE cFWE	7	4.9	2.9	136.5	49.0	3.10	2.35	3.10	2.35	0.63
	Mixed Effects	Fixed Effects MA	7	22.9	4.7	189.0	50.8	12.19	3.98	2.36	1.41	0.10
		Random Effects MA	7	21.4	3.4	169.2	44.0	10.38	3.22	1.40	0.94	0.07
		ALE Uncorrected	7	49.1	8.1	54.0	14.1	30.57	7.03	4.26	2.31	0.09
		ALE cFWE	7	11.6	1.5	147.3	26.1	5.95	1.77	5.95	1.77	0.52
20	Fixed Effects	Fixed Effects MA	3	19.3	5.1	438.1	145.5	8.00	4.29	2.17	1.83	0.12
		Random Effects MA	3	17.0	1.7	394.8	71.7	4.67	1.86	0.50	0.55	0.03
		ALE Uncorrected	3	52.3	8.4	128.1	42.0	22.67	7.37	4.67	2.16	0.09
		ALE cFWE	3	21.0	2.6	264.8	53.4	3.33	2.07	3.33	2.07	0.16
	OLS	Fixed Effects MA	3	21.3	8.7	248.9	105.2	12.33	7.55	3.17	0.75	0.15
		Random Effects MA	3	20.3	8.4	251.0	97.0	11.00	7.40	3.00	0.63	0.15
		ALE Uncorrected	3	47.0	11.1	64.9	17.3	29.00	9.72	5.17	2.14	0.11
		ALE cFWE	3	12.3	1.5	181.6	36.9	5.33	1.97	5.33	1.97	0.44
	Mixed Effects	Fixed Effects MA	3	20.7	4.5	389.8	122.1	8.67	4.27	1.00	1.10	0.05
		Random Effects MA	3	21.0	1.0	318.6	44.0	9.00	0.89	1.00	0.89	0.05
		ALE Uncorrected	3	50.7	6.1	123.9	36.5	26.67	5.16	5.67	2.80	0.11
		ALE cFWE	3	18.3	2.5	279.4	54.4	5.33	2.07	5.33	2.07	0.29
35	Fixed Effects	Fixed Effects MA	2	14.50	2.12	735.33	193.06	9.50	2.12	3.50	2.12	0.25
		Random Effects MA	2	12.50	3.54	793.10	308.16	4.50	3.54	2.00	1.41	0.17
		ALE Uncorrected	2	54.50	0.71	182.37	5.91	21.50	0.71	6.50	4.95	0.12
		ALE cFWE	2	25.50	0.71	347.01	37.16	4.50	0.71	4.50	0.71	0.17
	OLS	Fixed Effects MA	2	14.00	2.83	587.50	167.23	7.00	2.83	1.50	0.71	0.11
		Random Effects MA	2	13.50	2.12	600.95	144.08	6.50	2.12	1.50	0.71	0.11
		ALE Uncorrected	2	41.00	8.49	148.97	31.85	20.00	8.49	4.00	0.00	0.10
		ALE cFWE	2	15.50	0.71	350.41	9.69	3.50	0.71	3.50	0.71	0.22
	Mixed Effects	Fixed Effects MA	2	19.50	4.95	566.47	229.15	12.50	4.95	3.50	3.54	0.18
		Random Effects MA	2	17.50	3.54	578.73	203.84	9.50	3.54	3.00	2.83	0.17
		ALE Uncorrected	2	56.00	7.07	182.71	45.69	22.00	7.07	5.50	0.71	0.10
		ALE cFWE	2	22.00	2.83	402.95	27.23	5.00	2.83	5.00	2.83	0.23

*For each study set size (K), I replicated images are compared pairwise. Shown in the table are the averages (over I) of the amount of clusters and the size of these clusters. Next to it are the averages (over $\frac{I\;\times \;\left(I\;-\;1\right)\;}{2}$ pairwise comparisons) of the amount of clusters that are unique to one of the paired comparisons, the amount of large (i.e., more than 50 voxels) unique clusters and the percentage of the total amount of clusters that are large unique clusters*.

Regarding ALE, we clearly observe smaller regions of activation with a higher percentage of large unique clusters compared to the fixed and random effects meta-analysis, especially in small *K*. However, we do observe convergence in the ALE results to the brain regions characterized by (1) consistent statistically significant declared voxels (Figures 11B, 12B, 13B) and (2) high effect sizes in the reference images (Figures 11C, 12C, 13C). The fixed and random effects meta-analyses do detect larger regions, but are not necessarily constrained to the exact spatial shape of activated regions observed in the reference images.

The difference in the degree of unique information between uncorrected ALE and ALE cFWE is more detailed than the observed overlap values. Uncorrected ALE is associated with the highest (out of any meta-analysis) detection rate of small clusters. This in turn leads to an inflated number of (small and large) unique clusters. However, we observe the highest percentages of large unique clusters using ALE cFWE. Only small differences between the fixed and random effects meta-analyses are observed.

Regarding the group level models, we see on average less and smaller clusters of statistically significant voxels associated with the OLS group level models compared to the fixed and mixed effects models. This is true for every study set size *K*. However, for small study set sizes such as *K* = 10 and 20, the OLS model is associated with a higher percentage of large unique clusters. For *K* = 35, this is the opposite as the OLS model has on average the lowest percentage of large unique clusters. The fixed and mixed effects group level models show in most cases similar values. We include the distributions of the number of overlapping and unique detected clusters as well as the cluster sizes in section 7 in the Supplementary Material. These distributions show the same patterns as depicted in Table 3.

To conclude, models such as the OLS group level model (for *K* = 10 and 20) and the ALE meta-analyses that are characterized with low overlap values are either associated with smaller clusters of statistically significant voxels or higher percentages of large unique clusters.

### Between study variability

We observe no substantial differences between the fixed and random effects meta-analysis in most results. Since we are working with one large database of a homogenous sample executing the same paradigm, between study variability is limited. To illustrate, we refer to section 8 in the Supplementary Material depicting the distributions across all voxels of between-study variability and within-study variability, averaged over all folds.

**Figure 14:**
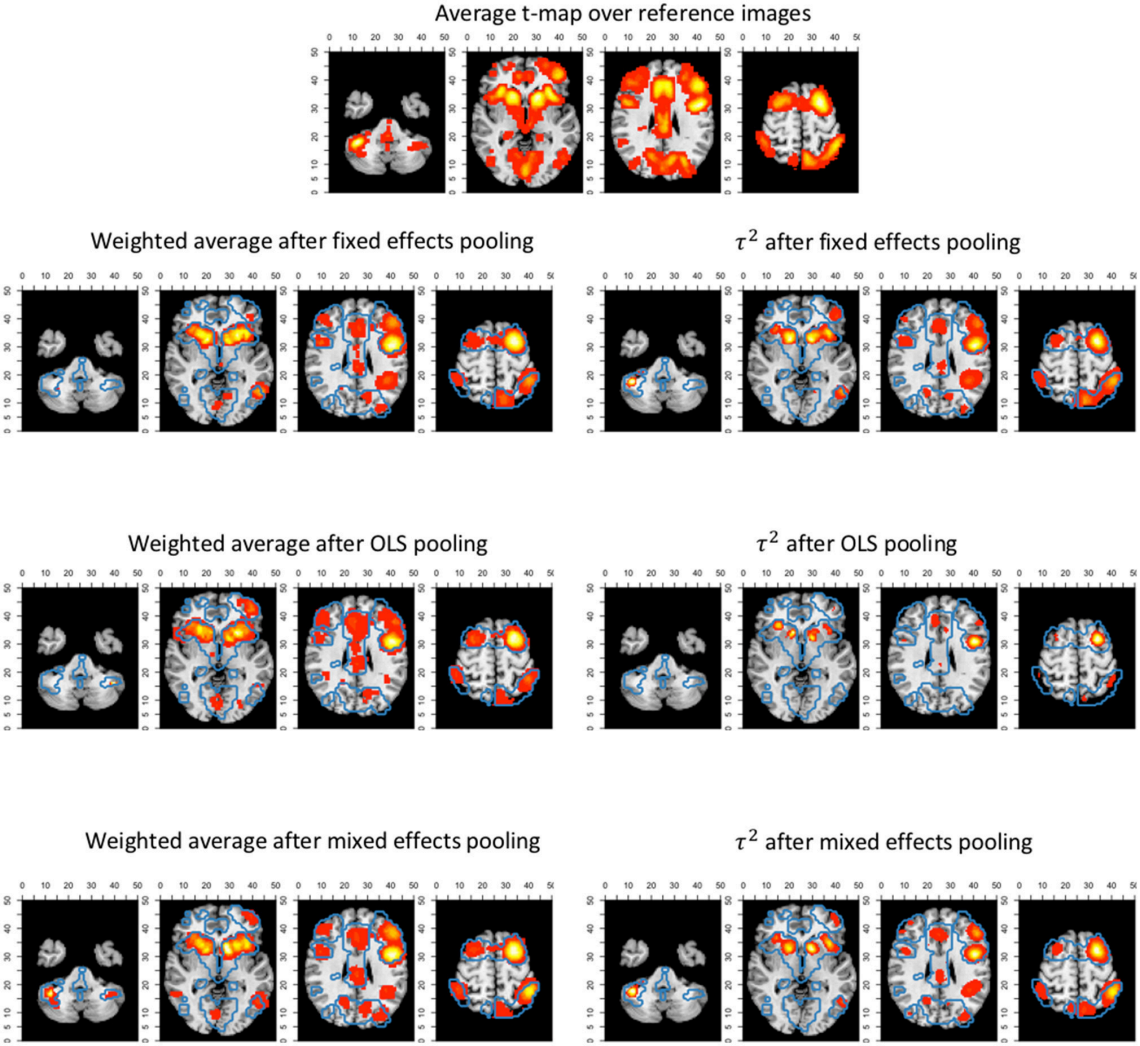
Slices (MNI z-coordinates from left to right: −44, −4, 26, and 58) showing the average t-map of the reference images, the estimated variance between studies and the weighted average of the random effects meta-analysis (statistically significant voxels only) using the 3 pooling models for *K* = 10. The contour lines represent the average t-map of the reference images shown as illustration.

To investigate this further, we look at the between study variability, estimated by τ^2^ in the weights (*U*_*vm*_ in Equation 10) of the random-effects meta-analysis for *K* = 10. In Figure 14, we display the average *t*-map (over 7-folds) of the reference images over 4 slices along the z-axis. We then plot the estimated τ^2^ from the random effects meta-analyses combined with the statistically significant voxels depicting the weighted averages of the random effects meta-analysis.

We observe the higher levels of between study heterogeneity mostly in the same regions that are statistically significant in the random (and fixed) effects meta-analysis (Figure 14). OLS pooling generates less between study heterogeneity compared to fixed and mixed effects pooling. This corresponds to the overall smaller differences in performance between fixed and random effects meta-analysis we observe when using OLS pooling (e.g., see Figures 6, 10).

## Discussion

In this paper, we studied how (1) the balance between false and true positives and (2) activation reliability for various coordinate-based meta-analysis (CBMA) methods in fMRI is influenced by an analytic choice at the study level. We applied a resampling scheme on a large existing dataset (*N* = 1,400) to create a test condition and an independent evaluation condition. Each test condition corresponds to a combination of (a) a method for pooling subjects within studies and (b) a meta-analytic method for pooling studies. For (a), we considered OLS, fixed effects and mixed effects modeling in FSL and for (b) we considered an activation likelihood estimation (ALE), a fixed effects coordinate-based meta-analysis and a random effects coordinate-based meta-analysis. We generated meta-analyses consisting of 10–35 studies. The evaluation condition corresponded to a high-powered image that was used as a reference outcome for comparison with the meta-analytical results.

Comparing the test and evaluation condition enabled to calculate false and true positive hits of the meta-analyses depicted in ROC curves for each specific combination. By resampling within test conditions, we explored various measures of reliability.

In our study, we found the most optimal balance between false and true positives when combining a mixed effects group level model with a random effects meta-analysis. For <20 studies in the meta-analyses, adding more studies lead to a better balance for this analysis pipeline. When the meta-analysis contained at least 20 studies, there was no further considerable improvement by adding studies. Our results further indicate that the combination of a random effects meta-analysis performed better with respect to activation reliability when combined with a fixed or mixed effects group level model. There are however two disadvantages when using fixed effects group level models. First, inference is restricted to the participants included in the study (Mumford and Nichols, 2006). Second, it has been shown that fixed effects models tend to be liberal (Mumford and Nichols, 2006). Hence, comparing two images with a large amount of positive hits (either be true or false positives) likely corresponds with an increased overlap.

Noticeably, the ROC curves demonstrate a worse balance between false and true positives when OLS group level models are used to pool subjects within studies, regardless of the meta-analysis. As shown in Mumford and Nichols (2009), OLS models tend to be associated with conservative hypothesis testing and a loss of power depending on the sample size and the extent to which the assumption of homogeneous within subject variability is violated (see also Friston et al., 2005). Our results are in line with Roels et al. (2016) who show favorable ROC curves in parametric testing of the mixed effects group level model compared to OLS.

Regarding the methods for CBMA, it can be noted that even though ALE only includes peak location and not peak height (effect size), results converge to the same brain regions associated with high effect sizes in the reference images. Subsequently, the ALE results tend to involve brain regions that correspond to the detected regions in the reference images. Eickhoff et al. (2016a) demonstrate that ALE meta-analyses require at least 20 studies to achieve a reasonable power. On the other hand, our results already indicate a relatively good balance between type I and II errors across the entire range of α when *K* = 10, using mixed effects group models. We do observe mostly higher values for the AUC using meta-analysis models relying on (standardized) effect sizes. As this difference is small, our findings differ from Radua et al. (2012), who observe much lower values for sensitivity when comparing ALE to seed based *d*-mapping. Their study was limited however to 10 studies per meta-analysis. Furthermore, these authors applied a FDR correction in ALE (at level 0.05) which is shown to be relatively low in sensitivity and susceptible to spurious activation for ALE maps (Eickhoff et al., 2016a). We on the other hand looked at a range of false positive rates given a significance level α which enables to study the power of procedures at an observed false positive rate.

However, we observed a considerably lower activation reliability when using ALE compared with the fixed and random effects methods for CBMA, even when 35 studies were included in the meta-analysis. We propose the following explanations. First in low study set sizes and as shown in Eickhoff et al. (2016a), ALE results that include only 10 studies are more likely to be driven by one single experiment. Second, the two approaches differ in the kernel sizes when modeling the foci. As described in Radua et al. (2012) and Eickhoff et al. (2009), the ALE algorithm relies on kernels with a smaller full-width at half maximum (FWHM) than the fixed and random effects meta-analyses. For the latter, the FWHM is validated at 20 mm (Radua et al., 2012). While for ALE, the FWHM is validated at 10 mm at most and decreases for studies with more subjects. This results in a greater number of small clusters of activation when using ALE. These images are more prone to be a hit or miss in a replication setting, depending on the sample size and the observed effect size. Furthermore, one expects to observe higher values of activation overlap between images with a higher amount of significantly activated voxels. Note that users can manually increase the FWHM. Third, the various methods use different approaches to correct for the multiple testing problem. For ALE we used the cFWE correction that was extensively validated in Eickhoff et al. (2016a). The fixed and random effects CBMA was implemented using the recommended thresholding of seed based *d*-mapping that relies on two (uncorrected) thresholds rather than explicitly correcting *P*-values. It remains unclear how this two-step thresholding procedure behaves in a range of scenarios where both the amount and location of peaks with respect to the true effect varies strongly.

We conclude with discussing some shortcomings of this paper. First, we did not investigate adaptive smoothing kernels such as the anisotropic kernel described in Radua et al. (2014). This type of kernel incorporates spatial information of the brain structure. These kernels are promising as they potentially result in a better delineation of the activated brain regions in a meta-analysis rather than the Gaussian spheres we observed in our results.

Second, our results are characterized by low between-study heterogeneity since each study is created by sampling from the same dataset. In a real meta-analysis, we expect higher between study variability as it will include studies with a range of different scanner settings, paradigm operationalizations, pre-processing pipelines (such as differences in normalization) and sample populations. In previous versions of this manuscript, we tested (1) sampling subjects in Figure 2 according to the scanning site involved in the IMAGEN project and (2) clustering subjects based on their individual effect size maps into individual studies to achieve higher between-study variability. However, these design adaptations did not yield substantial higher between-study heterogeneity. It should be noted that inference for fixed effects meta-analyses is restricted to the studies included in the meta-analysis. Random effects meta-analyses on the other hand allow for generalizations to the population (Borenstein et al., 2009). Furthermore the algorithm of ALE is developed in the spirit of random effects meta-analyses (Eickhoff et al., 2009).

Third, we limited our comparison to a fixed and random effects model implementation of an effect size based CBMA method with ALE, the most used CBMA method that only uses peak location. There are alternatives for ALE that also only use the location of local maxima such as Multilevel Kernel Density Analysis (Wager et al., 2007, 2009).

Fourth, we did not explicitly investigate the influence of the sample size of individual studies on the outcome of a meta-analysis. However, Tahmasebi et al. (2012) used the same IMAGEN dataset (though with a different contrast) to measure the effect of the sample size on the variability of the locations of peak activity in group analyses (study level). Their results indicate that 30 participants or more are needed so that locations of peak activity stabilize around a reference point. For similar results, see Thirion et al. (2007) who recommend at least 20 participants in a group analysis to achieve acceptable classification agreement. This was defined as the concordance between group analyses containing different subjects performing the same experimental design on declaring which voxels are truly active.

Finally, it should be stressed that our study does not reveal which combinations are more robust against the presence of bias. This bias could include (1) publication bias (Rothstein et al., 2005), (2) bias due to missing information since only statistically significant peak coordinates and/or peak effect sizes are used within studies and not the entire image, (3) or in the case of effect size based CBMA bias due to missing data if peak effect sizes for some studies are not reported (Wager et al., 2007; Costafreda, 2009). Seed based *d*-mapping, uses imputations to solve this latter missing data problem. As we did not have any missing data in our simulations, we did not evaluate the influence of these missing data on the performance of the various CBMA methods.

## Conclusion

There is a clear loss of information when fMRI meta-analyses are restricted to coordinates of peak activation. However, if complete statistical parametric maps are unavailable, then coordinate based meta-analyses provide a way to aggregate results. We have investigated the trajectory of fMRI results from the choice of statistical group models at the study level to different coordinate-based meta-analysis methods. Our results favor the combination of mixed effects models in the second stage of the GLM procedure combined with random effects meta-analyses which rely on both the coordinates and effect sizes of the local maxima. Our results indicated (1) a higher balance between the false and true positive rate when compared to a high-powered reference image and (2) a higher reliability if the meta-analysis contains at least 20 or 35 studies. The popular ALE method for coordinate-based meta-analysis provides a slightly lower but still comparable balance between false and true positives. However, it needs at least 35 studies to approach the higher levels of activation reliability associated with a random effects model for coordinate-based meta-analysis. The main advantage of our work consists of using a large database, while the main limitation is the restriction to only one dataset. We argue that this work provides substantial insight into the performance of coordinate based meta-analyses for fMRI.

## Author contributions

HB, RS, SK, and BM: contributed to the conception and design of the manuscript. Data collection and single subject analyses were carried out by the IMAGEN consortium represented by TB, GB, AB, J-LM, HL, TP, and SM. Data analysis and interpretation for this study was performed by HB, RS, and BM. Next, HB developed the initial draft of the manuscript. Finally, all authors approve the version to be published.

### Conflict of interest statement

TB has served as an advisor or consultant to Bristol-Myers Squibb, Desitin Arzneimittel, Eli Lilly, Medice, Novartis, Pfizer, Shire, UCB, and Vifor Pharma; he has received conference attendance support, conference support, or speaking fees from Eli Lilly, Janssen McNeil, Medice, Novartis, Shire, and UCB; and he is involved in clinical trials conducted by Eli Lilly, Novartis, and Shire; the present work is unrelated to these relationships. GB has received funding for a Ph.D. student and honoraria from General Electric for teaching on scanner programming courses from General Electric Healthcare; he acts as a consultant for IXICO. The other authors declare that the research was conducted in the absence of any commercial or financial relationships that could be construed as a potential conflict of interest.

## Footnotes

^1^http://i2bm.cea.fr/drf/i2bm/english/Pages/NeuroSpin/Presentation.aspx

^2^http://www.fil.ion.ucl.ac.uk/spm/software/spm8/

^3^http://adni.loni.usc.edu/methods/documents/mri-protocols/

^4^http://www.sdmproject.com
