# Supplementary material for: The Influence of Study-Level Inference Models and Study Set Size on Coordinate-Based fMRI Meta-Analyses
Source: Front Neurosci. 2018 Jan 18;11:745. doi: 10.3389/fnins.2017.00745 (PMC5778144; doi:10.3389/fnins.2017.00745)

# *Supplementary Material*

# The Influence of Study-Level Inference Models and Study Set Size on Coordinate-Based fMRI Meta-Analyses.

**Han Bossier[1]*; Ruth Seurinck Ph.D. [1]; Simone Kühn Ph.D. [2]; Tobias Banaschewski M.D., Ph.D.[3]; Gareth J. Barker Ph.D.[4]; Arun L.W. Bokde Ph.D.[5]; Jean-Luc Martinot M.D., Ph.D.[6]; Herve Lemaitre Ph.D.[7]; Tomáš Paus M.D., Ph.D.[8]; Sabina Millenet Dipl.-Psych.[3] and Beatrijs Moerkerke Ph.D. [1]**

*** Correspondence:** Han Bossier: Han.Bossier@Ugent.be

## 1 ROC curves for all values of *K*

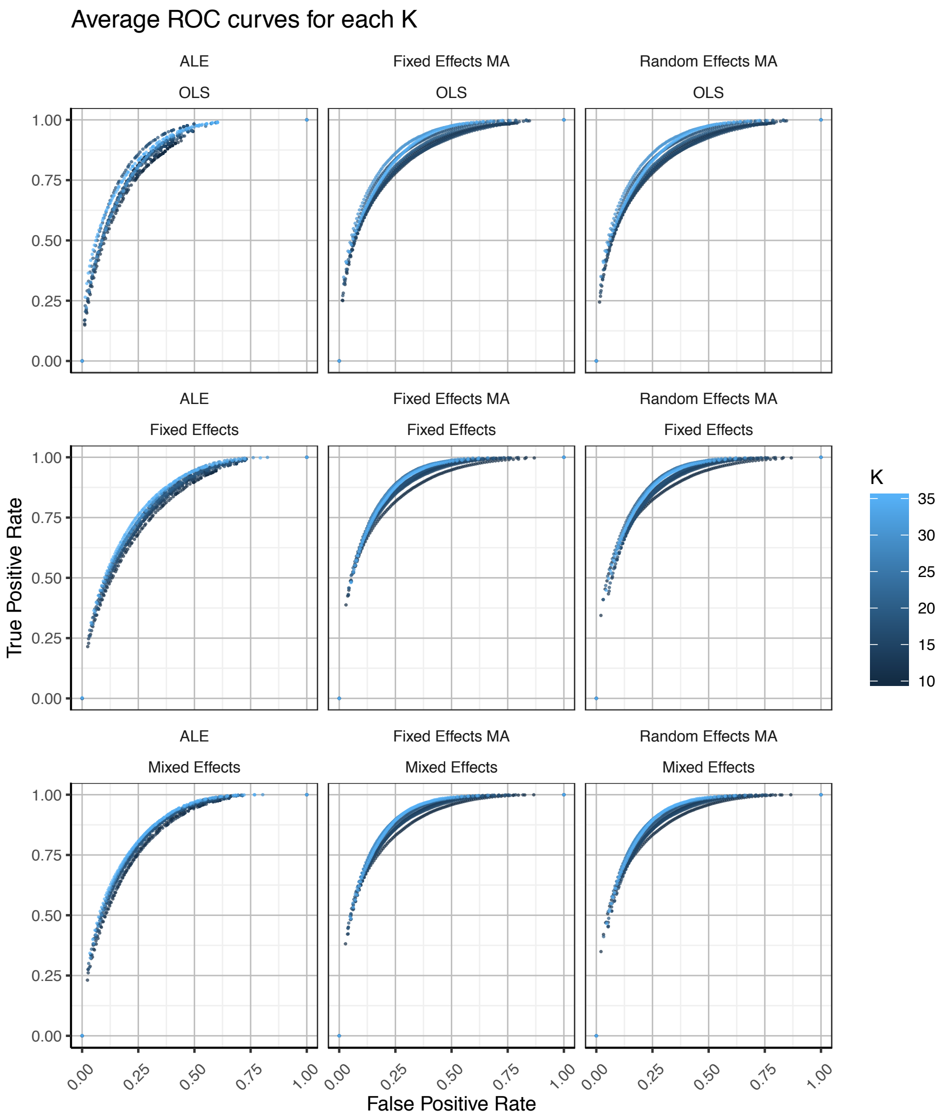

## 2 Partial ROC plots when $\boldsymbol{\alpha} \in [\mathbf{0}, \mathbf{0.1}]$.

For $K$ = 10, 20 and 35, we plot partial ROC curves between α ∈ [0,0.1]. The values in each pane correspond to the partial Area Under the Curves. The drop-down lines correspond to the point at which the pre-specified nominal level is set at an uncorrected α level of 0.05.

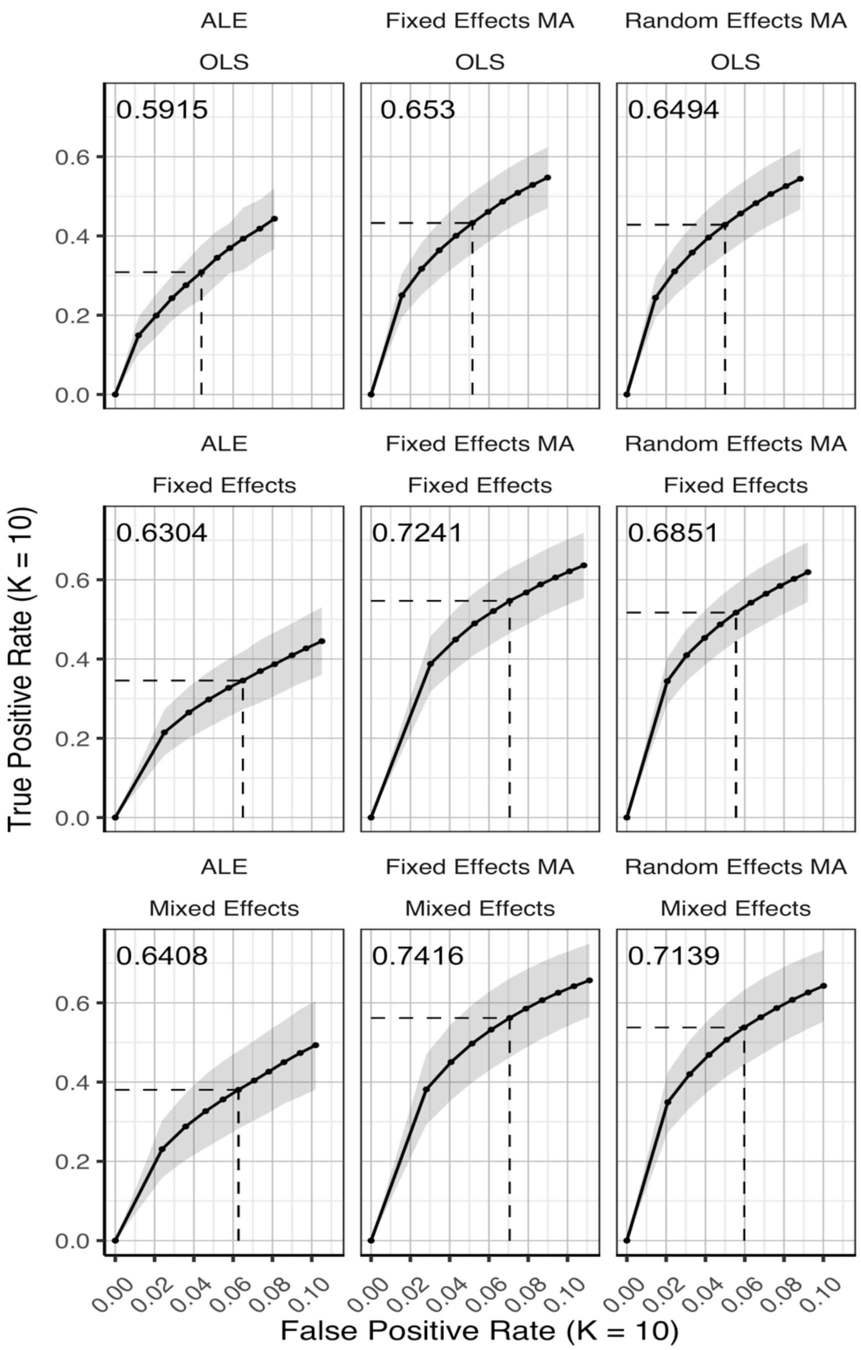

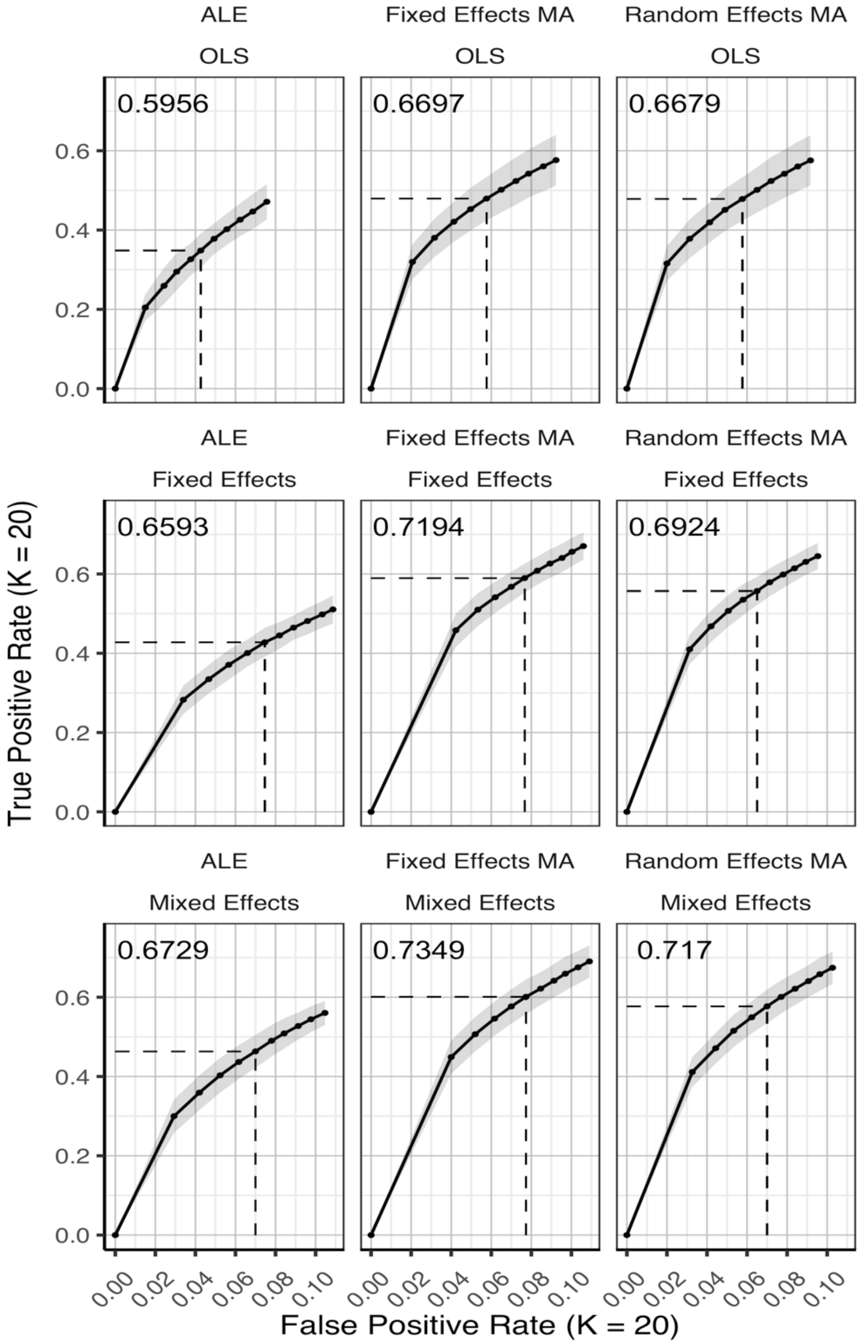
ALE
OLS
0.5956
Fixed Effects MA
OLS
0.6697
Random Effects MA
OLS
0.6679
ALE
Fixed Effects
0.6593
Fixed Effects MA
Fixed Effects
0.7194
Random Effects MA
Fixed Effects
0.6924
ALE
Mixed Effects
0.6729
Fixed Effects MA
Mixed Effects
0.7349
Random Effects MA
Mixed Effects
0.717
True Positive Rate (K = 20)
0.0
0.2
0.4
0.6
0.00
0.02
0.04
0.06
0.08
0.10
False Positive Rate (K = 20)

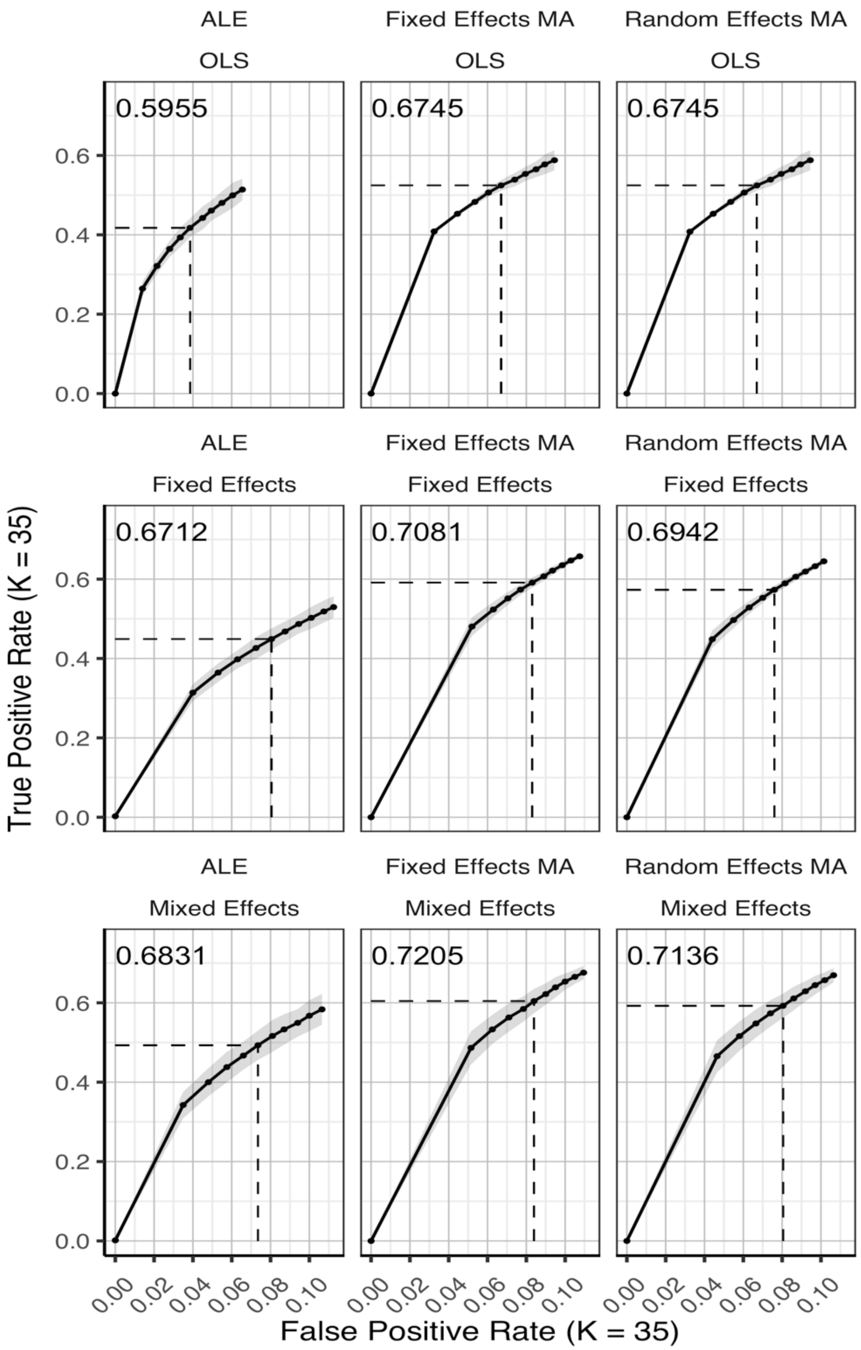

ALE
OLS
0.5955
Fixed Effects MA
OLS
0.6745
Random Effects MA
OLS
0.6745
ALE
Fixed Effects
0.6712
Fixed Effects MA
Fixed Effects
0.7081
Random Effects MA
Fixed Effects
0.6942
ALE
Mixed Effects
0.6831
Fixed Effects MA
Mixed Effects
0.7205
Random Effects MA
Mixed Effects
0.7136
0.0
0.2
0.4
0.6
True Positive Rate (K = 35)
0.00
0.02
0.04
0.06
0.08
0.10
False Positive Rate (K = 35)

## 3 ROC plots using reference images with FDR control at level 0.05

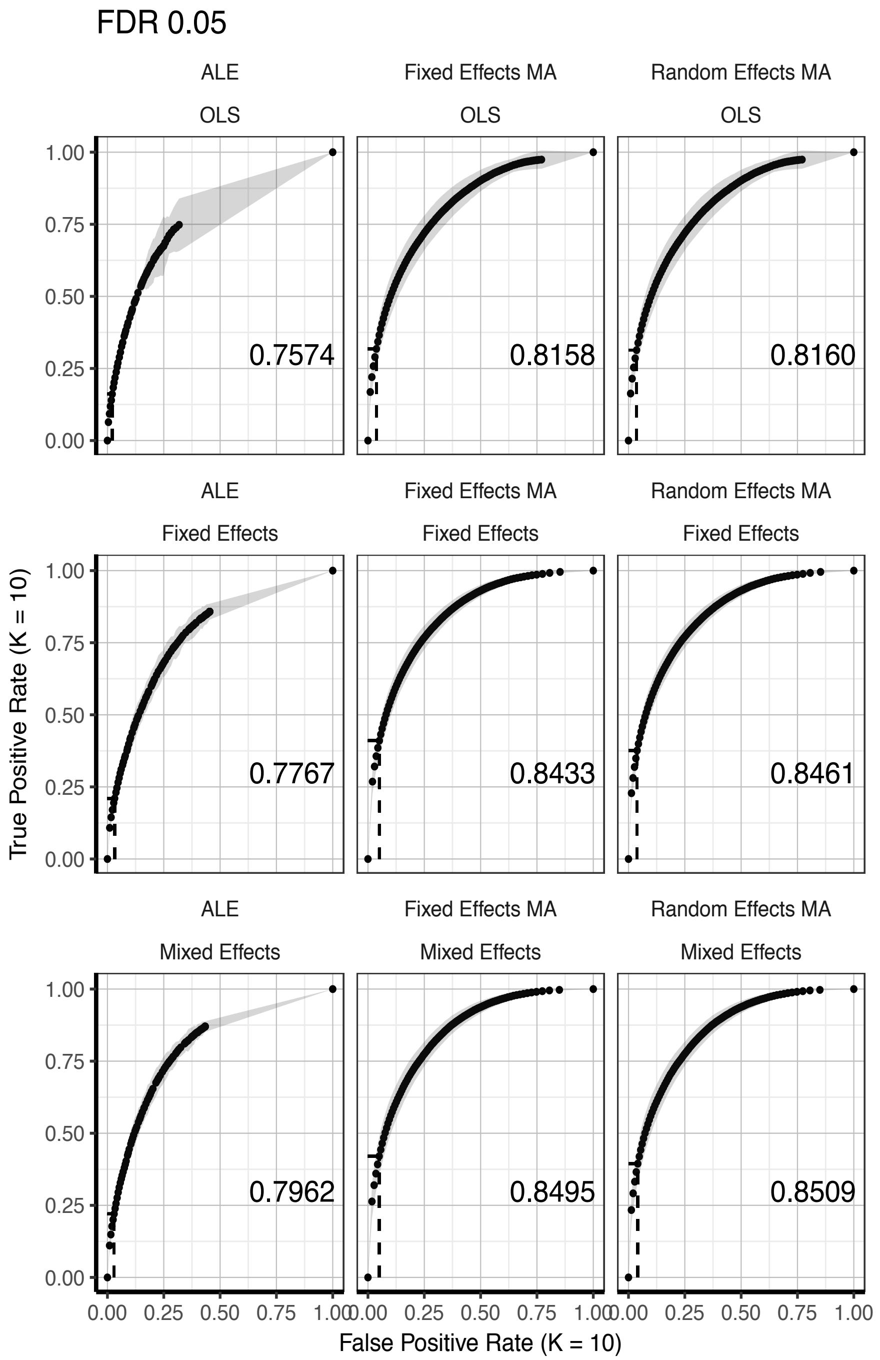

FDR 0.05

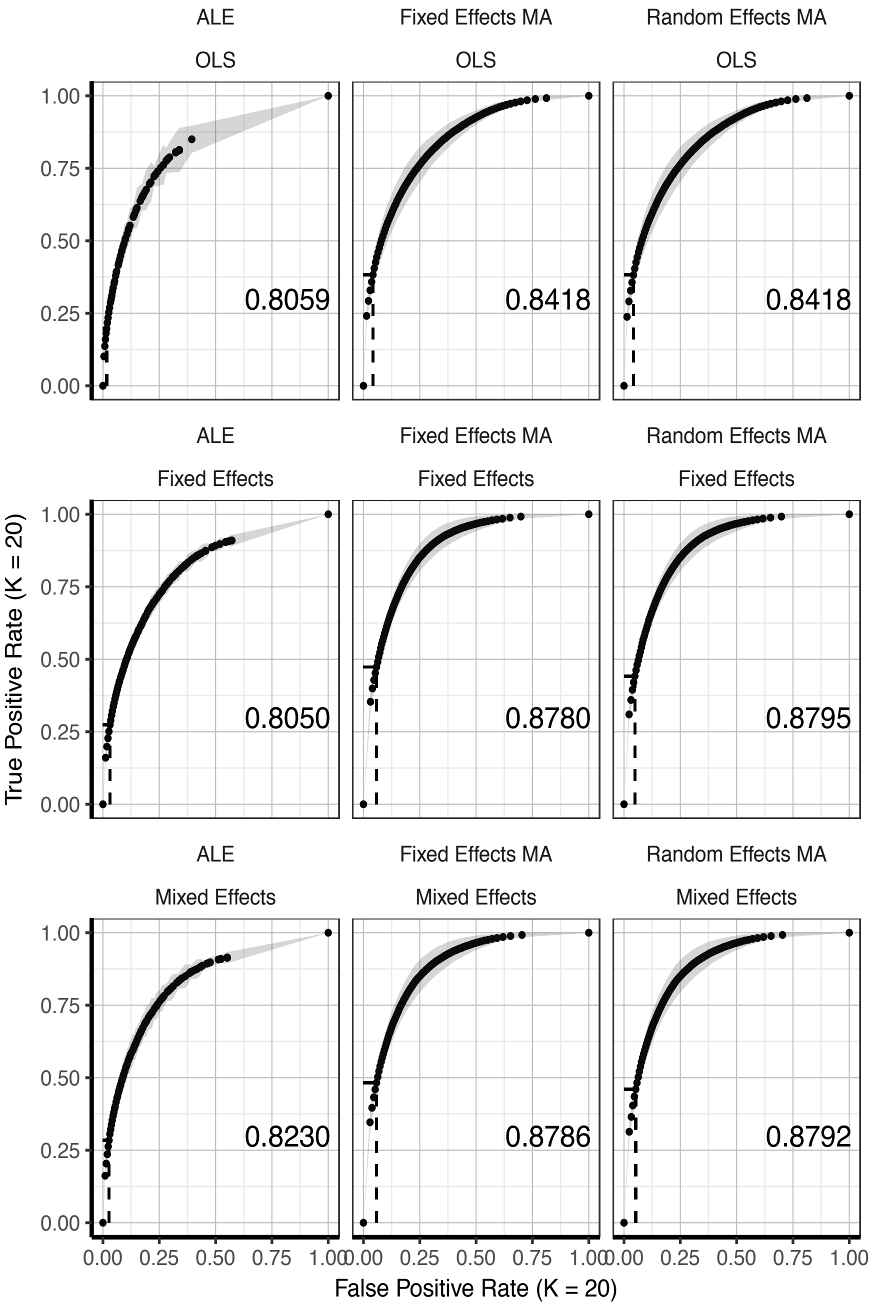
ALE
OLS
Fixed Effects MA
OLS
Random Effects MA
OLS
0.8059
0.8418
0.8418
ALE
Fixed Effects
Fixed Effects MA
Fixed Effects
Random Effects MA
Fixed Effects
0.8050
0.8780
0.8795
ALE
Mixed Effects
Fixed Effects MA
Mixed Effects
Random Effects MA
Mixed Effects
0.8230
0.8786
0.8792
True Positive Rate (K = 20)
False Positive Rate (K = 20)
0.00
0.25
0.50
0.75
1.00

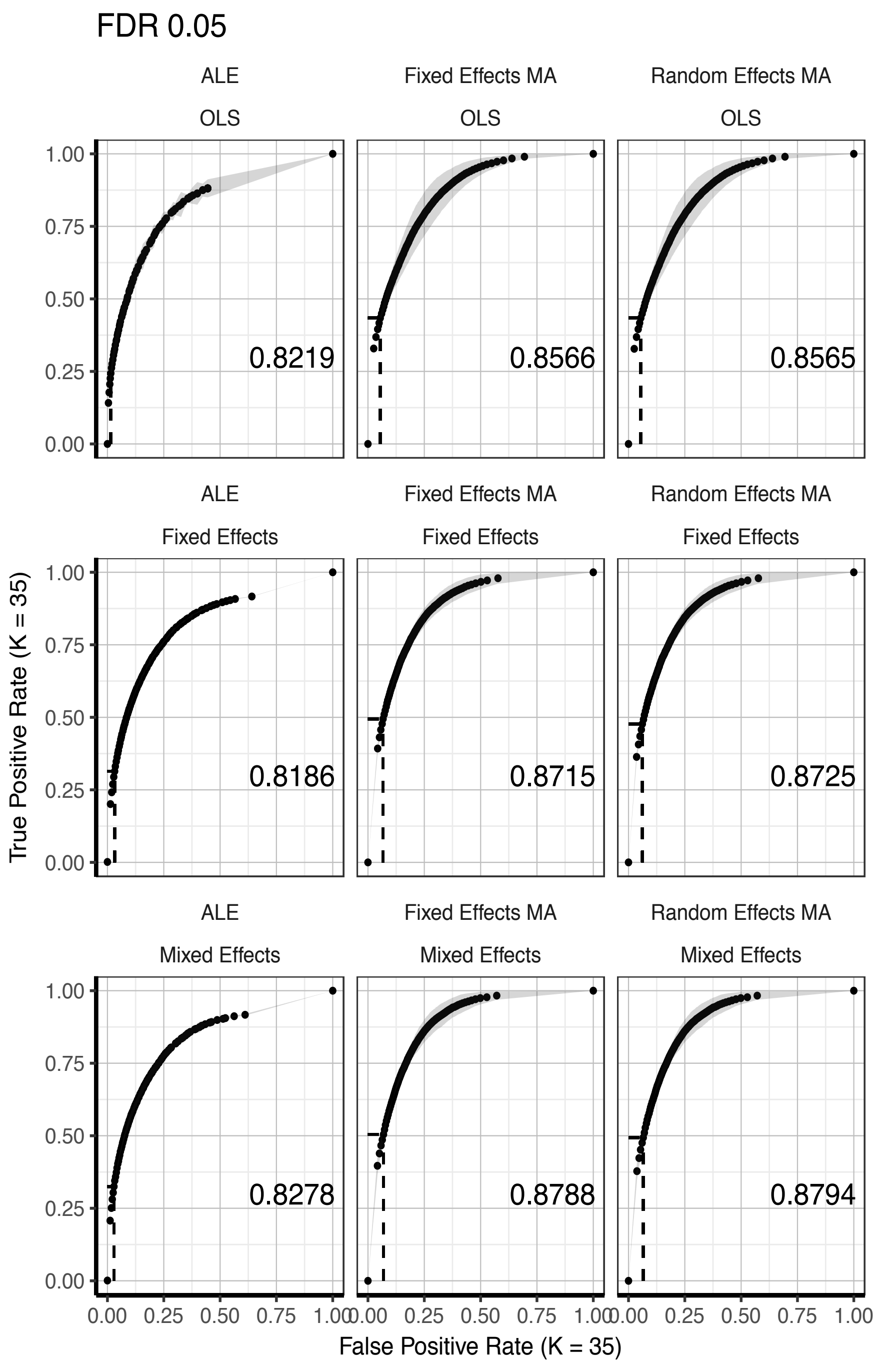

FDR 0.05
ALE
OLS
Fixed Effects MA
OLS
Random Effects MA
OLS
0.8219
0.8566
0.8565
ALE
Fixed Effects
Fixed Effects MA
Fixed Effects
Random Effects MA
Fixed Effects
0.8186
0.8715
0.8725
ALE
Mixed Effects
Fixed Effects MA
Mixed Effects
Random Effects MA
Mixed Effects
0.8278
0.8788
0.8794
True Positive Rate (K = 35)
False Positive Rate (K = 35)
0.00
0.25
0.50
0.75
1.00

# 4 ROC plots using reference images with FDR control at level 0.001

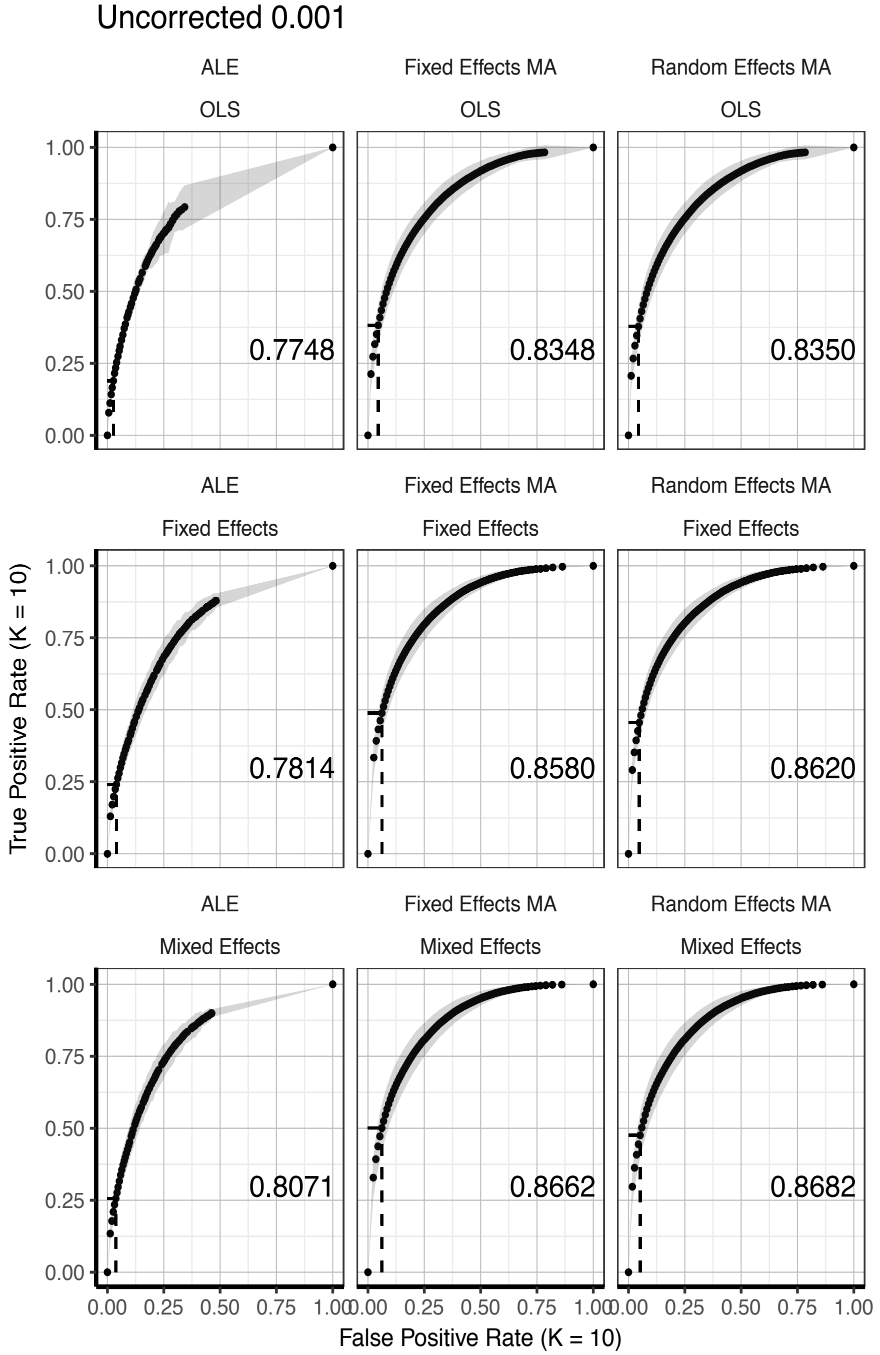

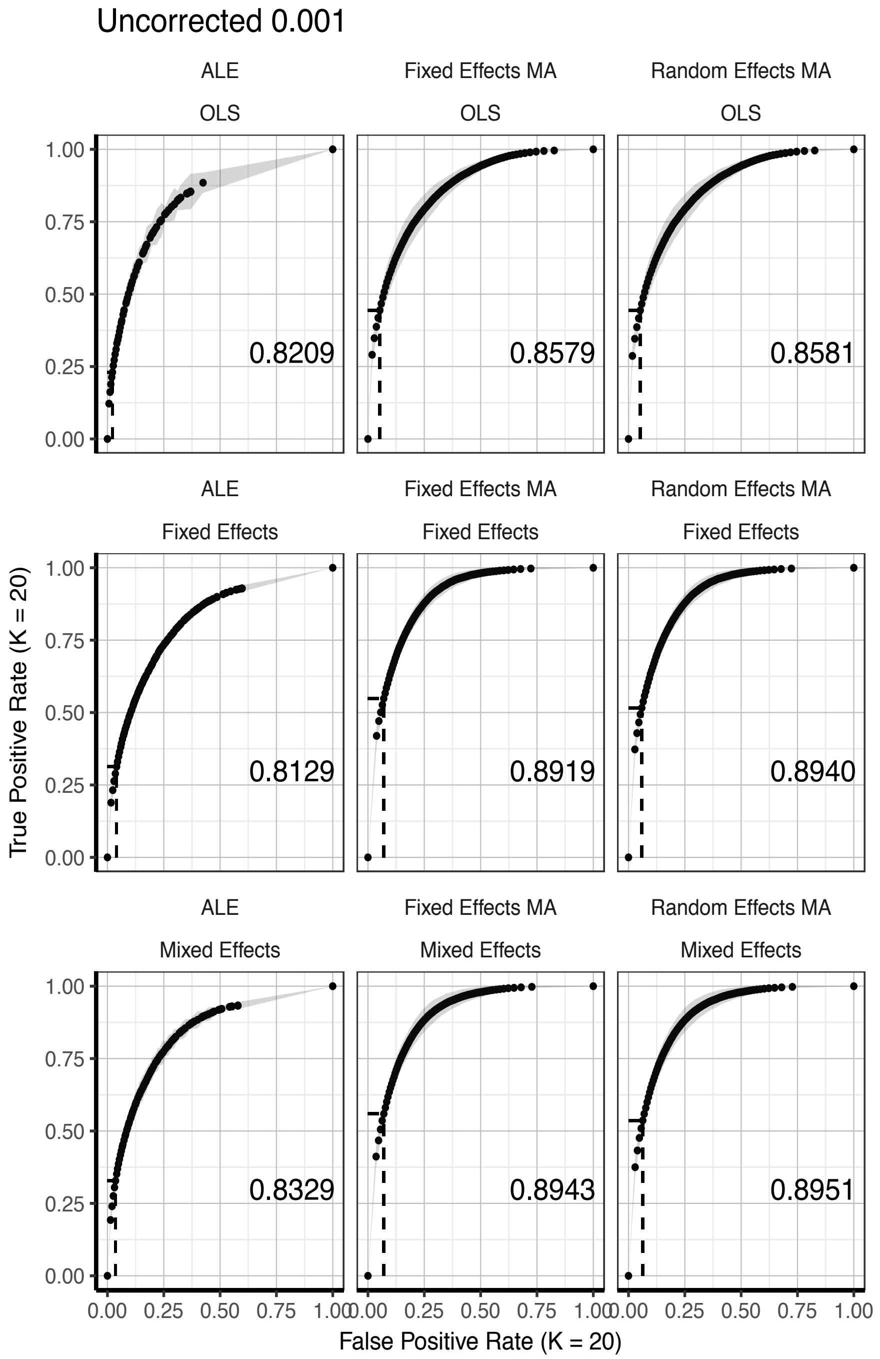
Uncorrected 0.001
ALE
OLS
Fixed Effects MA
OLS
Random Effects MA
OLS
0.8209
0.8579
0.8581
ALE
Fixed Effects
Fixed Effects MA
Fixed Effects
Random Effects MA
Fixed Effects
0.8129
0.8919
0.8940
ALE
Mixed Effects
Fixed Effects MA
Mixed Effects
Random Effects MA
Mixed Effects
0.8329
0.8943
0.8951
True Positive Rate (K = 20)
False Positive Rate (K = 20)
0.00
0.25
0.50
0.75
1.00

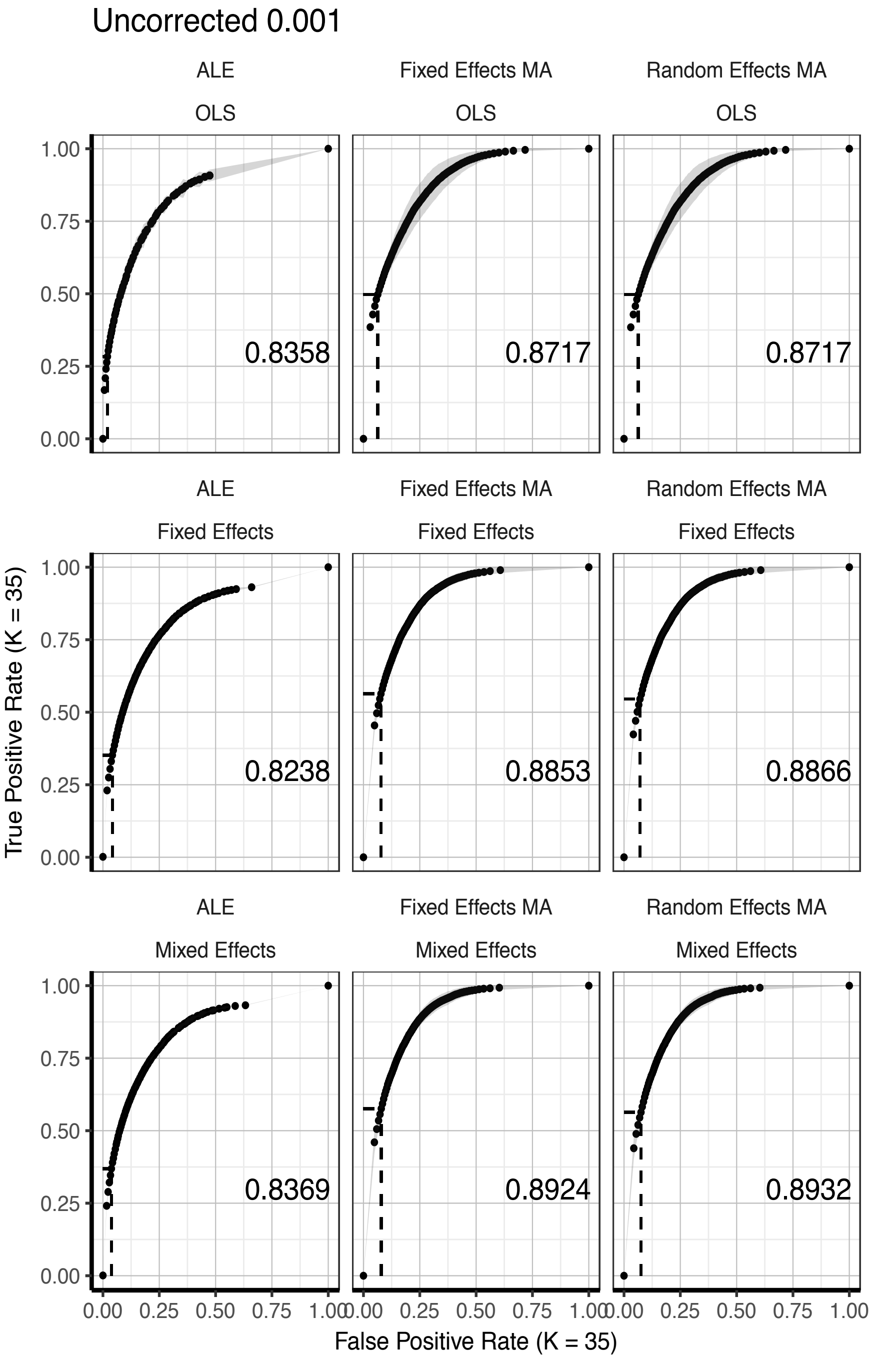
Uncorrected 0.001
ALE
OLS
Fixed Effects MA
OLS
Random Effects MA
OLS
0.8358
0.8717
0.8717
ALE
Fixed Effects
Fixed Effects MA
Fixed Effects
Random Effects MA
Fixed Effects
0.8238
0.8853
0.8866
ALE
Mixed Effects
Fixed Effects MA
Mixed Effects
Random Effects MA
Mixed Effects
0.8369
0.8924
0.8932
True Positive Rate (K = 35)
False Positive Rate (K = 35)
0.00
0.25
0.50
0.75
1.00

## 5 Percent overlap between thresholded meta-analyses with $K$ = 12, 14, 16, 18 and 30

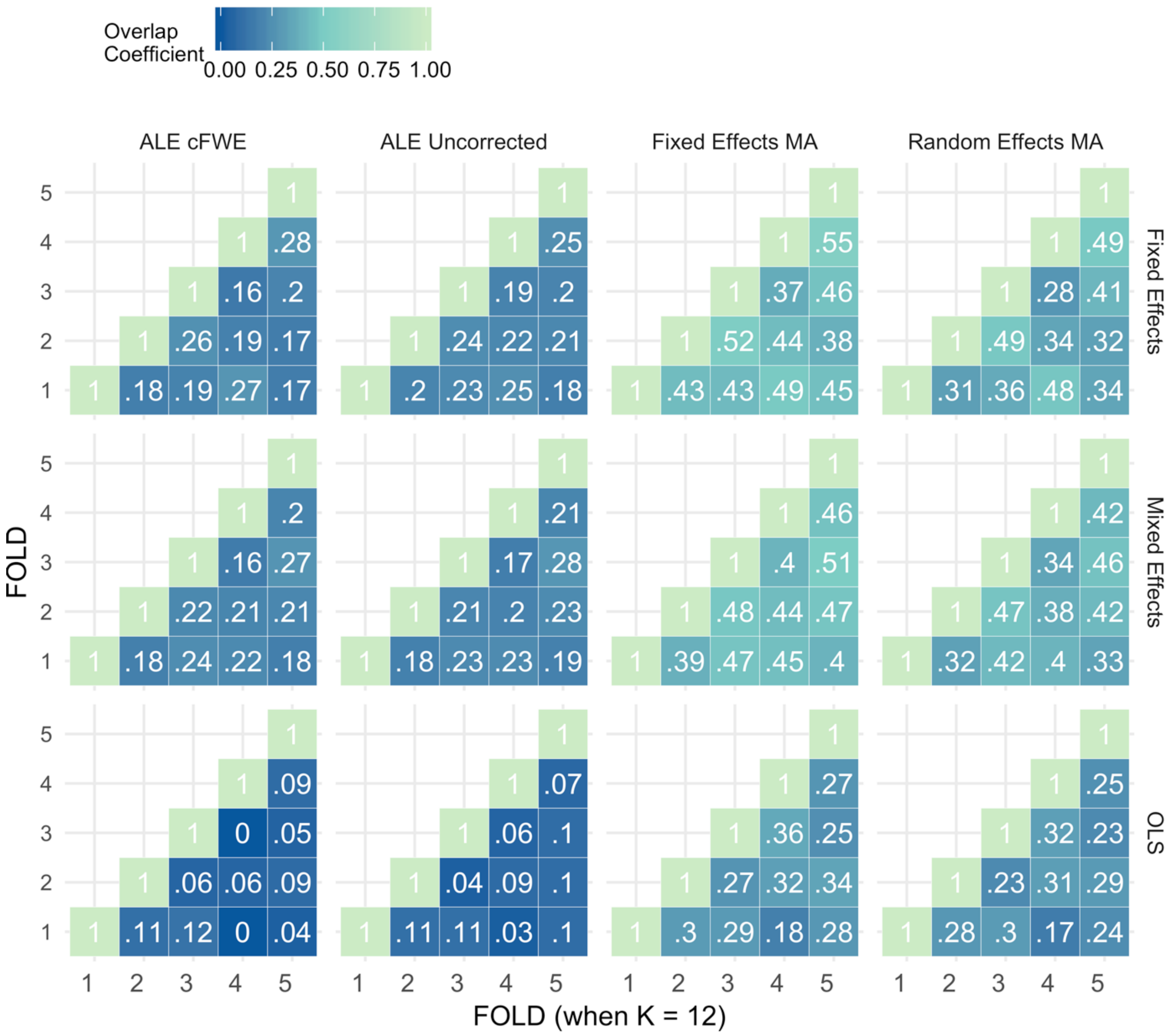

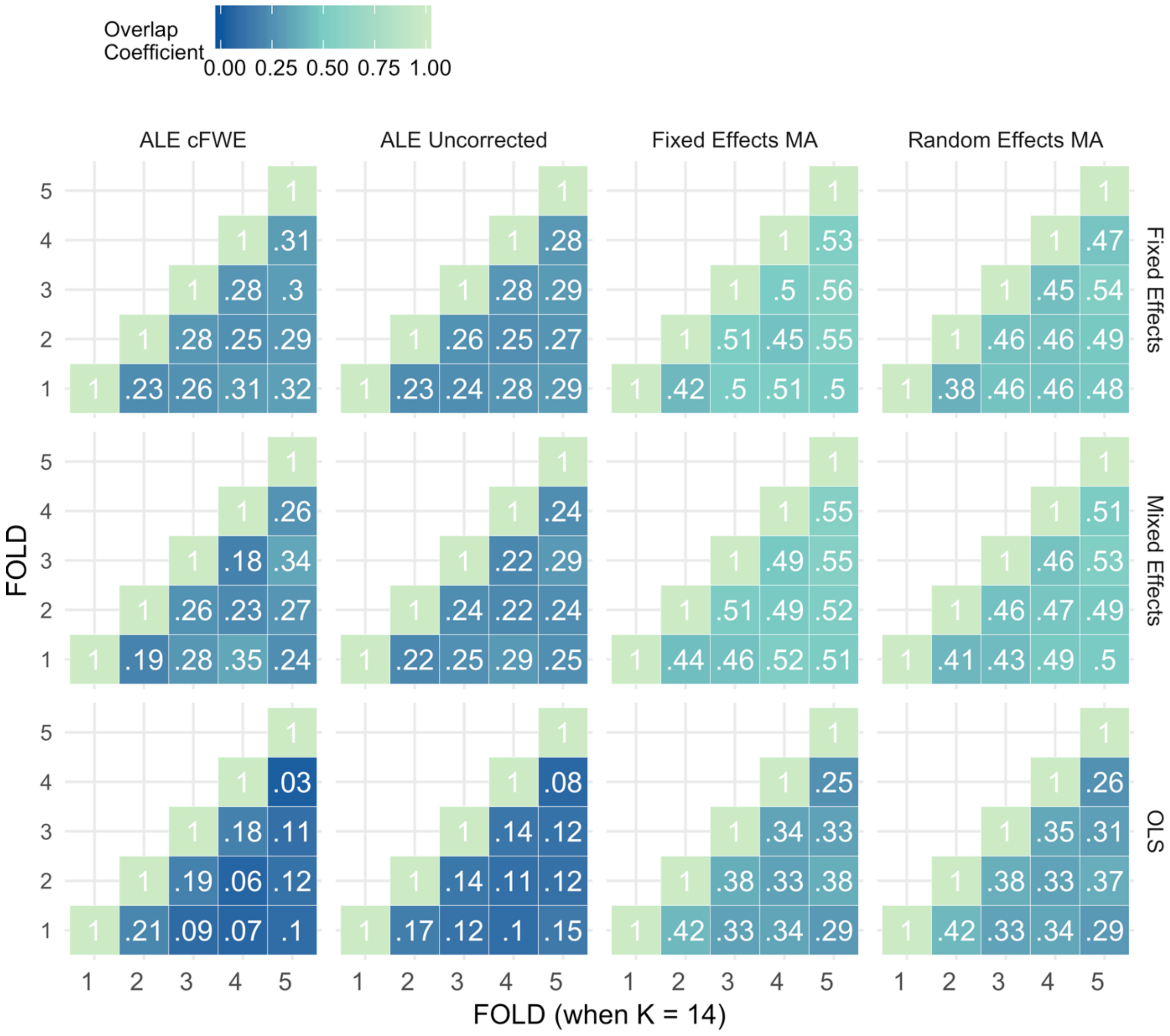
Overlap Coefficient
0.00 0.25 0.50 0.75 1.00
ALE cFWE
ALE Uncorrected
Fixed Effects MA
Random Effects MA
Fixed Effects
Mixed Effects
OLS
FOLD
FOLD (when K = 14)

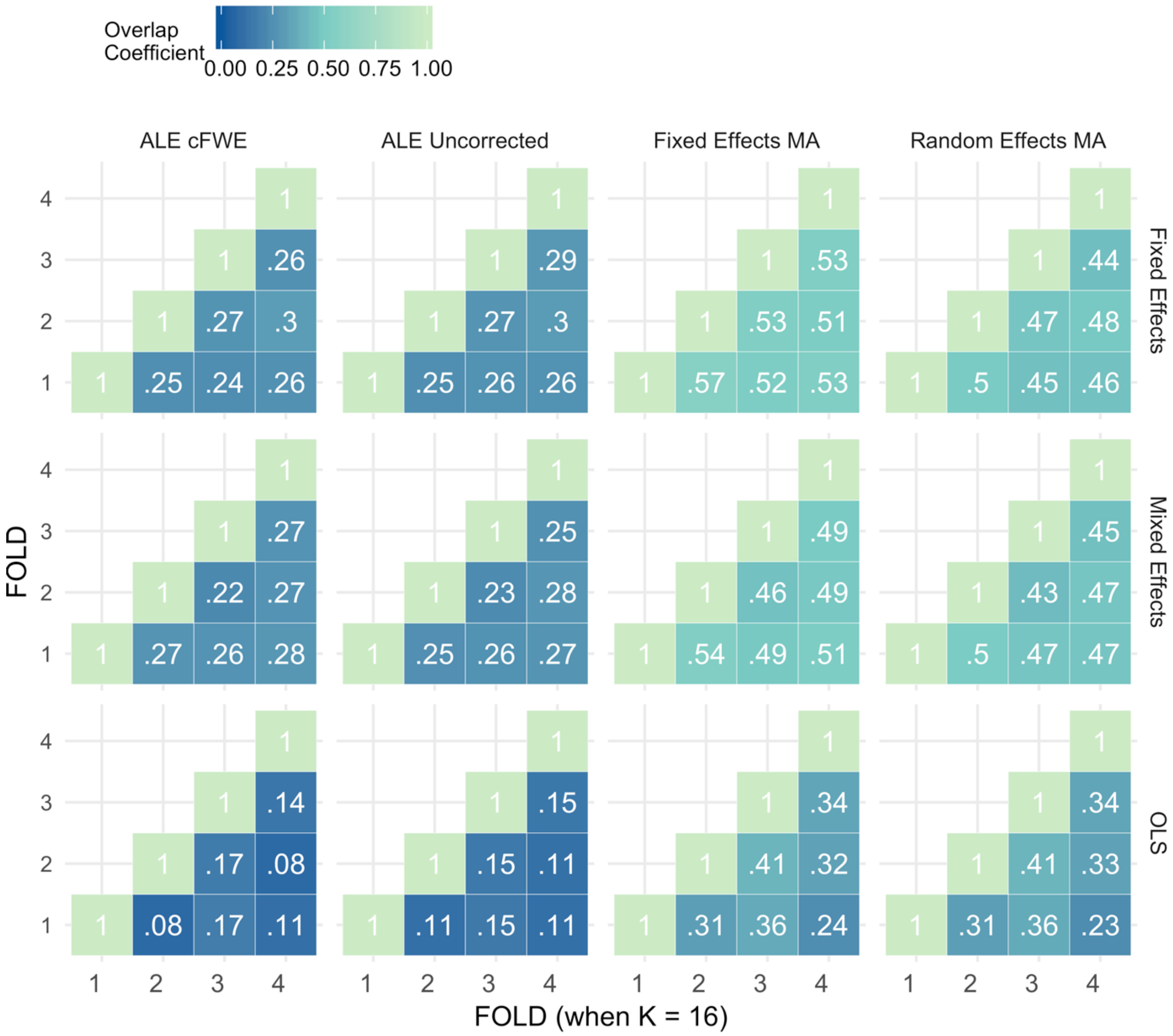

Overlap Coefficient
0.00 0.25 0.50 0.75 1.00
ALE cFWE
ALE Uncorrected
Fixed Effects MA
Random Effects MA
Fixed Effects
Mixed Effects
OLS
FOLD
FOLD (when K = 16)

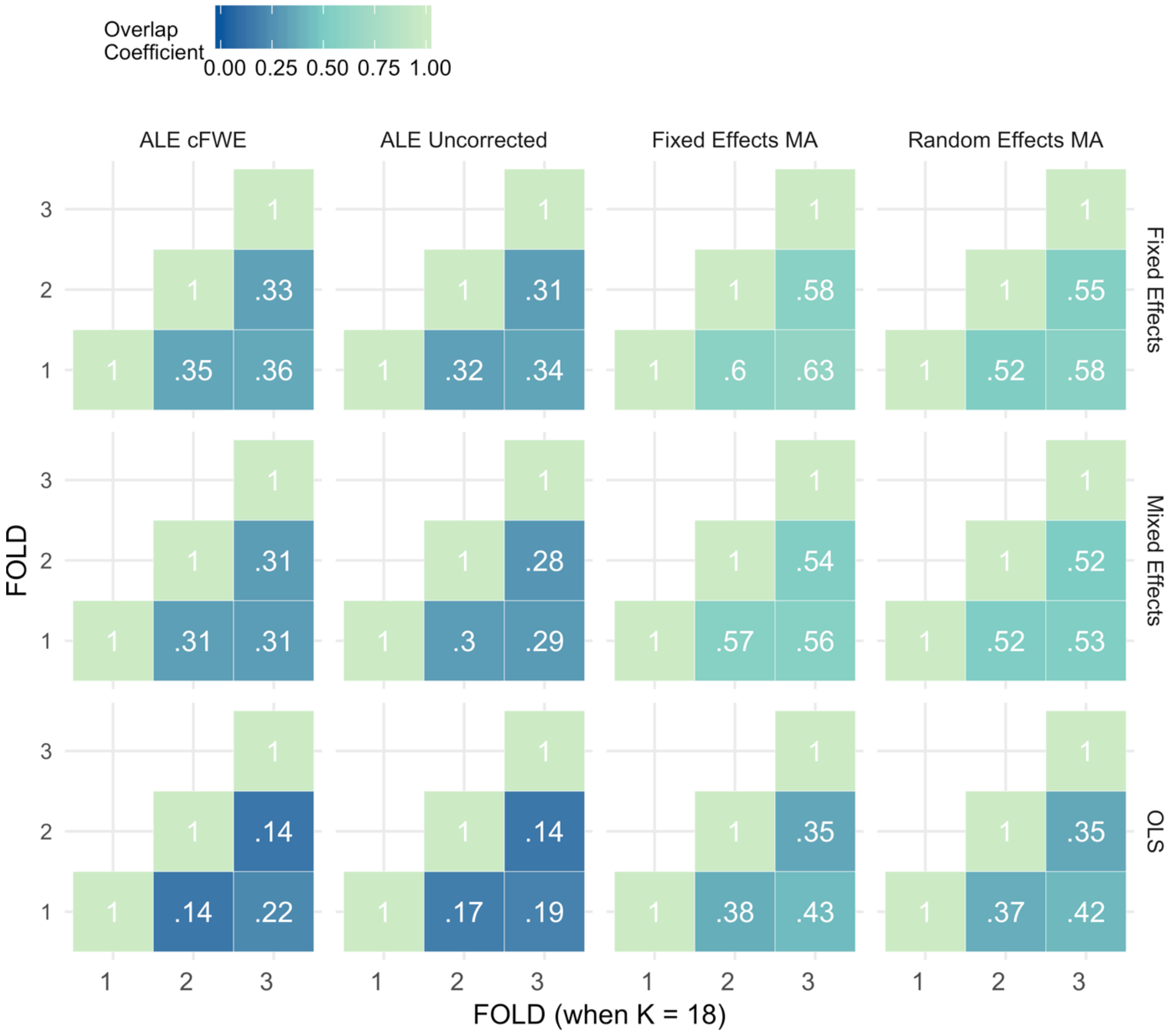
Overlap
Coefficient
0.00 0.25 0.50 0.75 1.00
ALE cFWE
ALE Uncorrected
Fixed Effects MA
Random Effects MA
Fixed Effects
Mixed Effects
OLS
FOLD
FOLD (when K = 18)
.35
.36
.33
.32
.34
.31
.6
.63
.58
.52
.58
.55
.31
.31
.31
.3
.29
.28
.57
.56
.54
.52
.53
.52
.14
.22
.14
.17
.19
.14
.38
.43
.35
.37
.42
.35

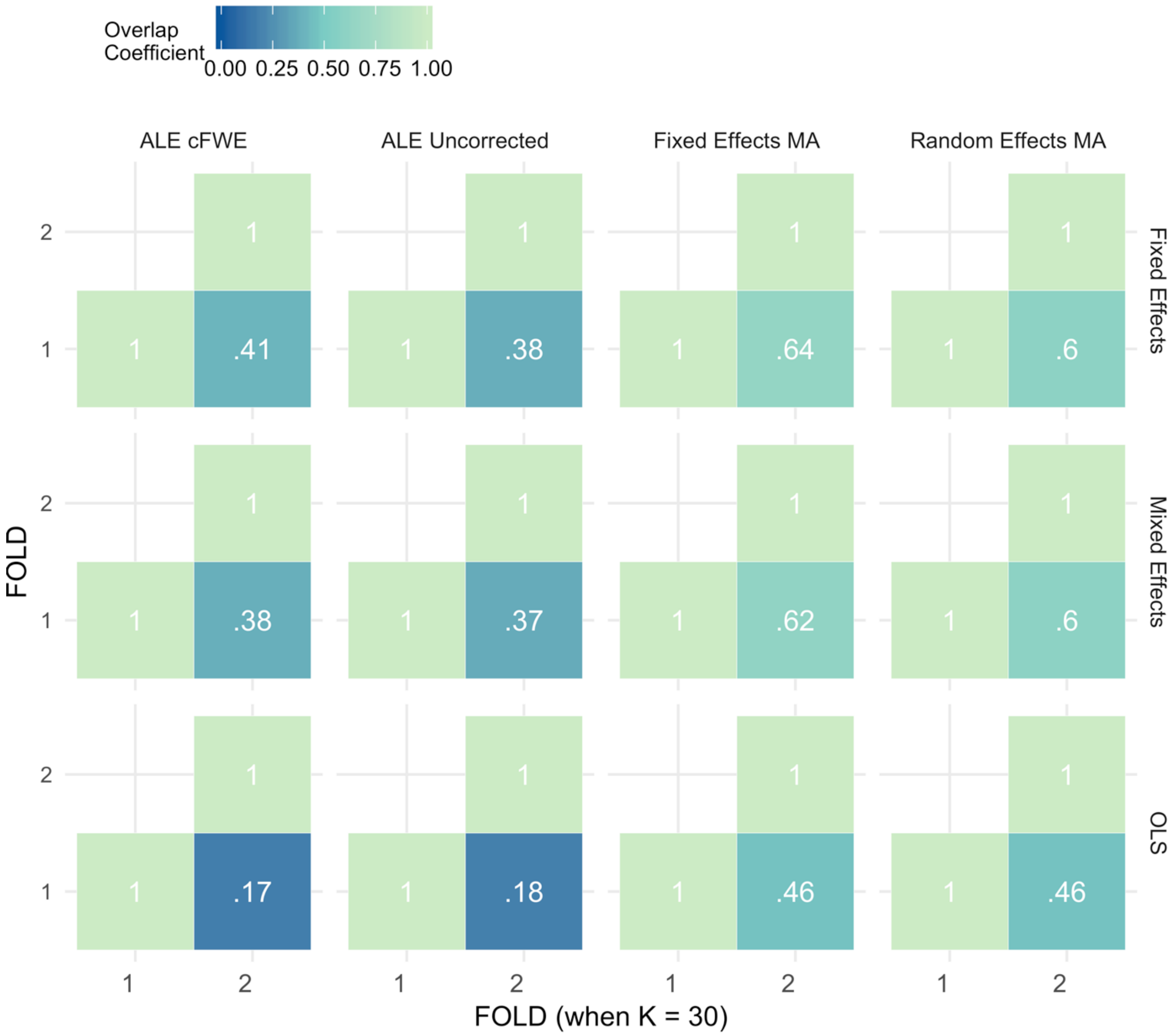
Overlap Coefficient
0.00 0.25 0.50 0.75 1.00
ALE cFWE
ALE Uncorrected
Fixed Effects MA
Random Effects MA
Fixed Effects
Mixed Effects
OLS
FOLD
FOLD (when K = 30)
.41
.38
.64
.6
.38
.37
.62
.6
.17
.18
.46
.46

## 6 Percent overlap for thresholded meta-analysis at P = 0.001.

In the following figure, we plot the average percent overlap between independent folds for ALE, the fixed and random effects CBMA while increasing *K*. For each method, we use a statistical threshold at P = 0.001, uncorrected for multiple testing.

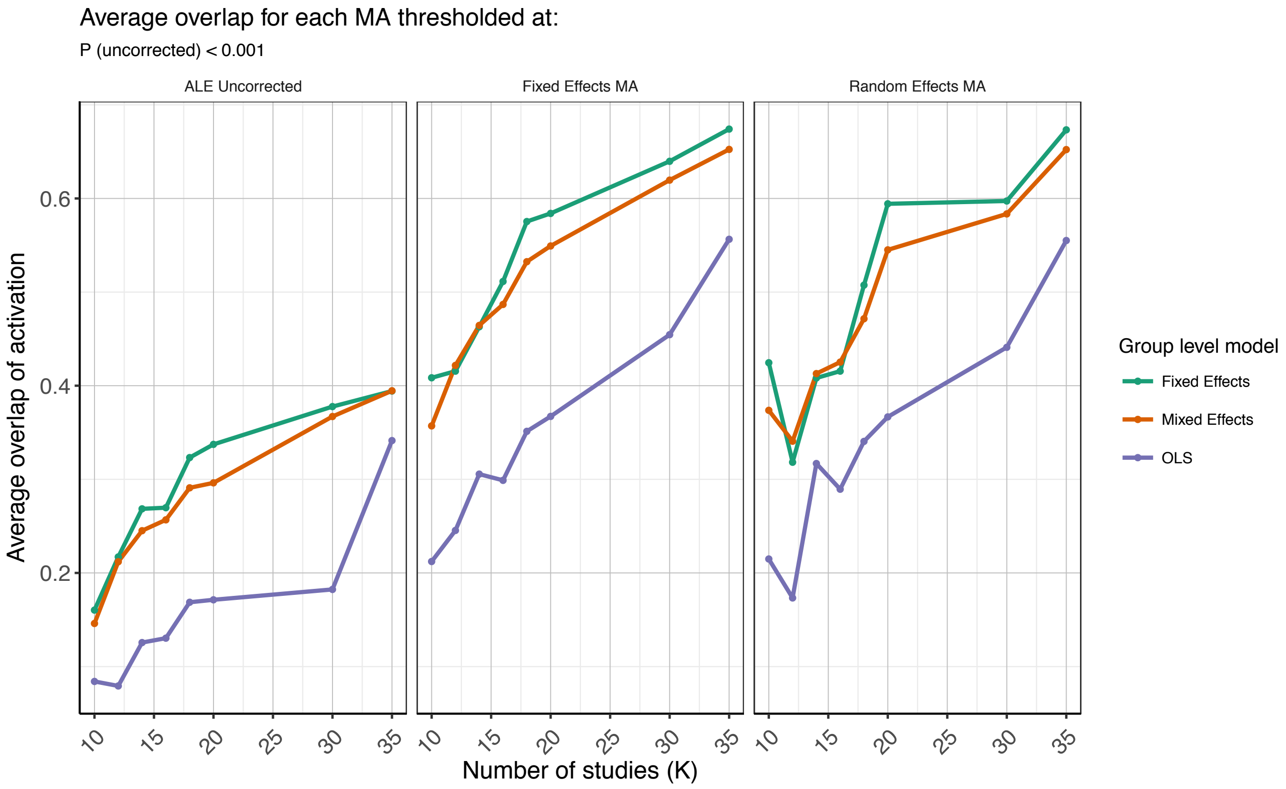

# 7 Distributions of amount and cluster sizes.

For $K$ = 10, 20 and 35, we plot the amount of overlapping and unique clusters with the cluster sizes (expressed in number of voxels) next to it. This is calculated on the pairwise comparisons of the I unique folds. We plot the results for each group level model and CBMA.

## 7.1 K = 10

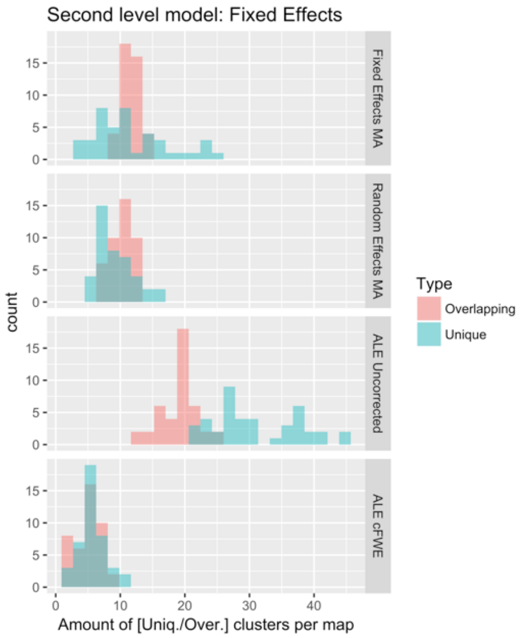


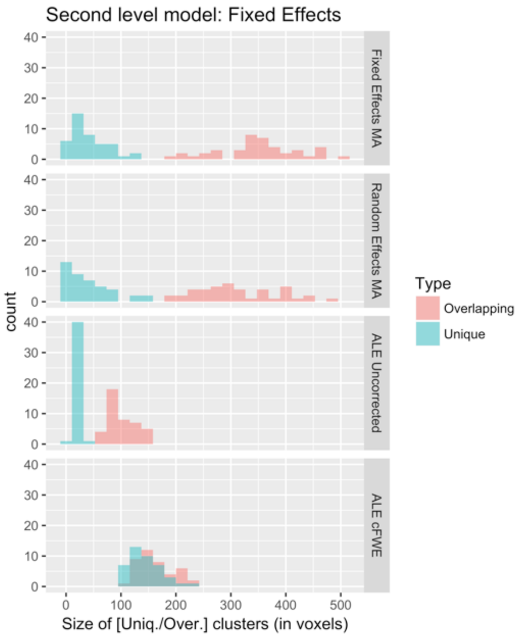


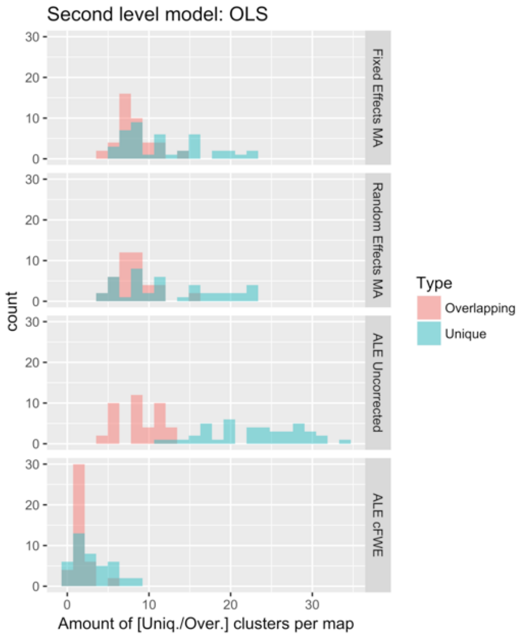


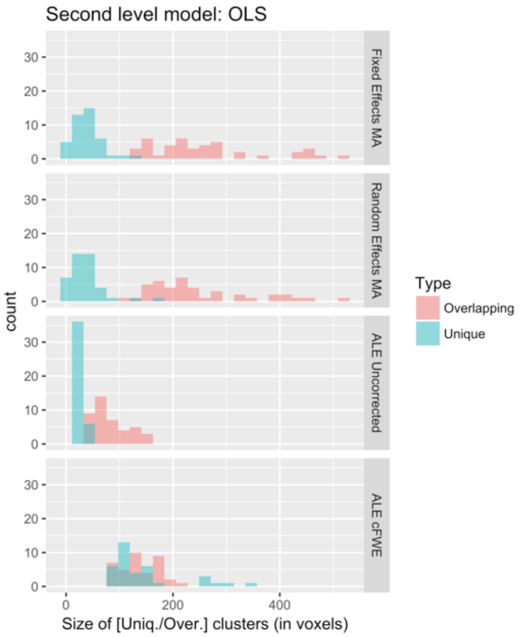

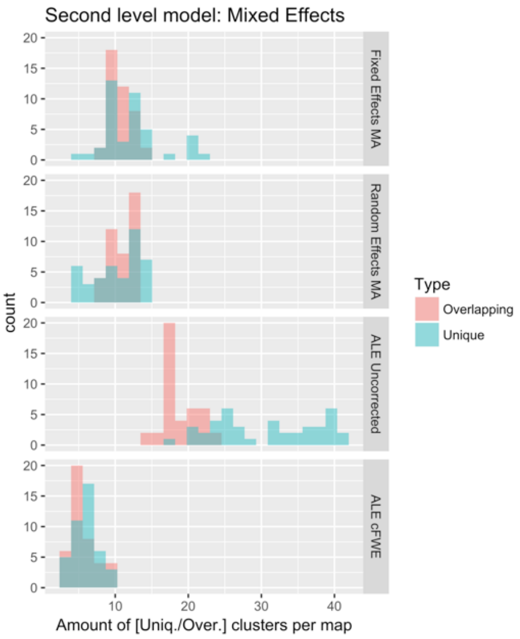
Second level model: Mixed Effects
Fixed Effects MA
Random Effects MA
ALE Uncorrected
ALE cFWE
count
Type
Overlapping
Unique
Amount of [Uniq./Over.] clusters per map

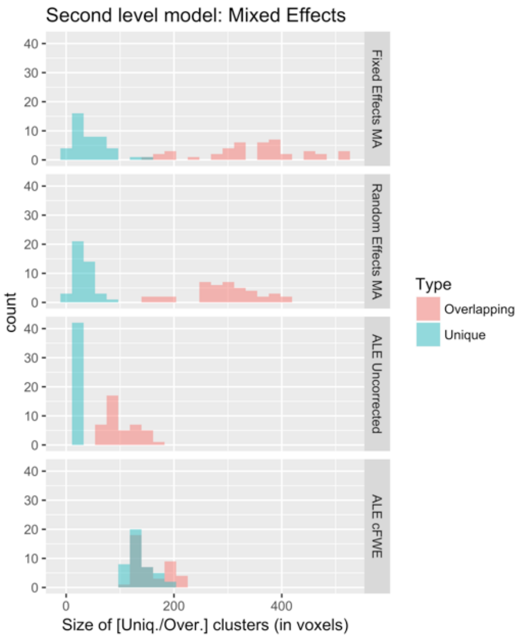
Second level model: Mixed Effects
Fixed Effects MA
Random Effects MA
ALE Uncorrected
ALE cFWE
count
Type
Overlapping
Unique
Size of [Uniq./Over.] clusters (in voxels)

## 7.2 K = 20

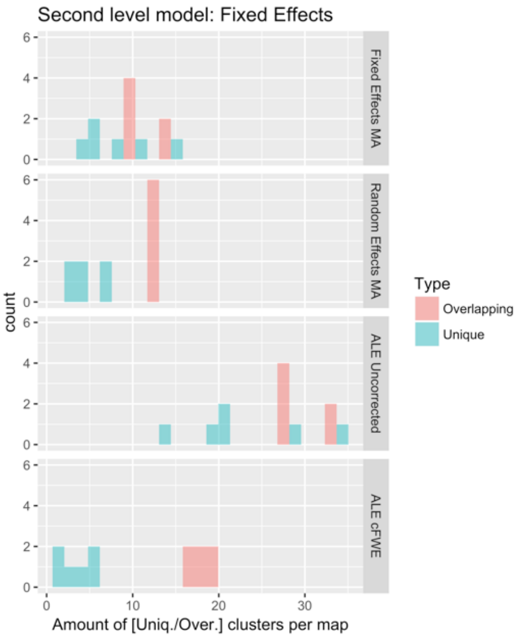
Second level model: Fixed Effects
Fixed Effects MA
Random Effects MA
ALE Uncorrected
ALE cFWE
count
Type
Overlapping
Unique
Amount of [Uniq./Over.] clusters per map

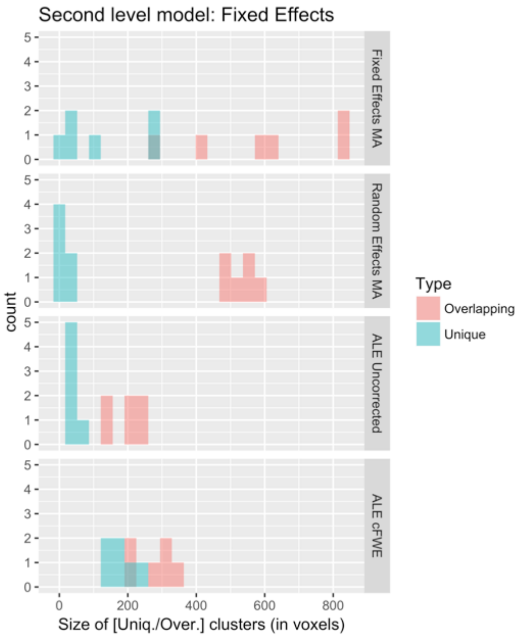
Second level model: Fixed Effects
Fixed Effects MA
Random Effects MA
ALE Uncorrected
ALE cFWE
count
Type
Overlapping
Unique
Size of [Uniq./Over.] clusters (in voxels)

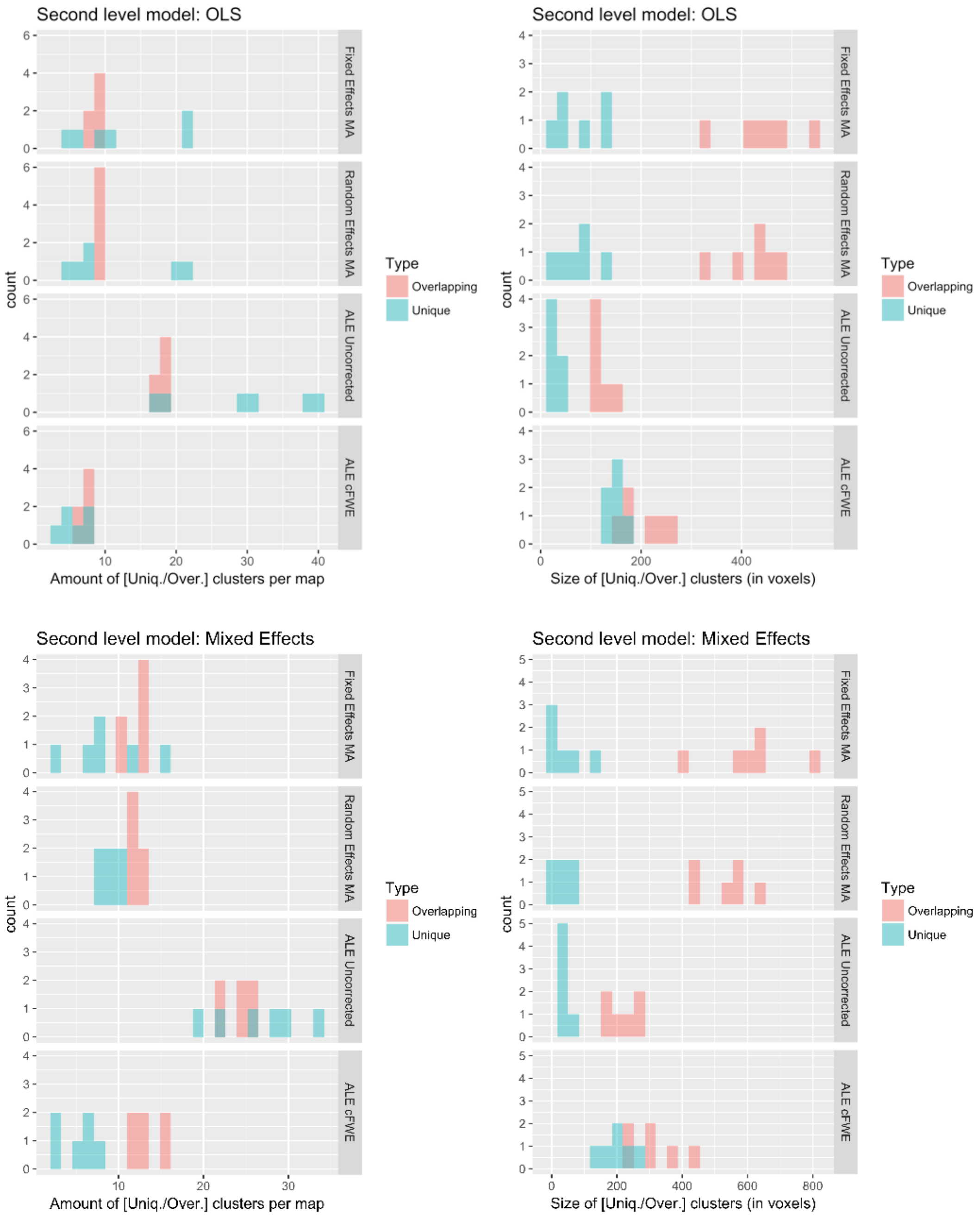
Second level model: OLS
Amount of [Uniq./Over.] clusters per map
Size of [Uniq./Over.] clusters (in voxels)
Second level model: Mixed Effects
count
Fixed Effects MA
Random Effects MA
ALE Uncorrected
ALE cFWE
Type
Overlapping
Unique

## 7.3 K = 35

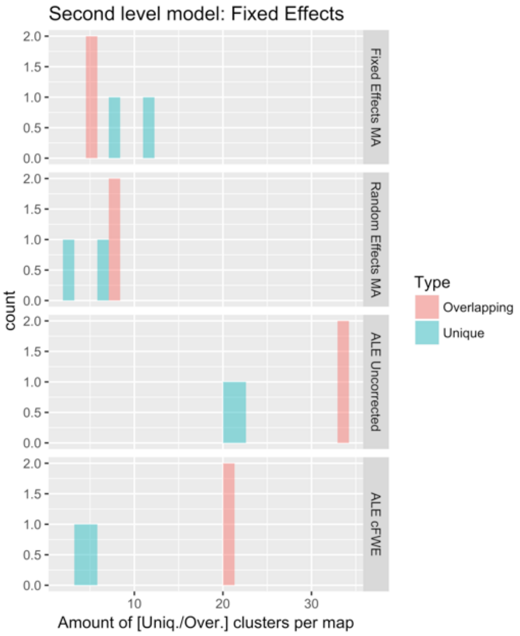
Second level model: Fixed Effects
count
Fixed Effects MA
Random Effects MA
ALE Uncorrected
ALE cFWE
Amount of [Uniq./Over.] clusters per map
Type
Overlapping
Unique

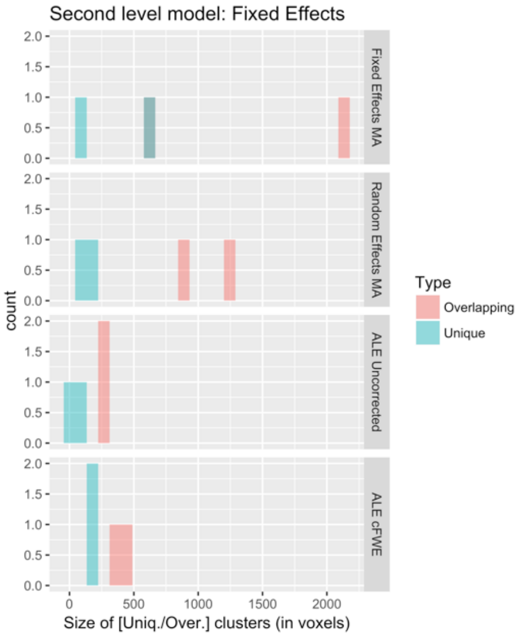
Second level model: Fixed Effects
count
Fixed Effects MA
Random Effects MA
ALE Uncorrected
ALE cFWE
Size of [Uniq./Over.] clusters (in voxels)
Type
Overlapping
Unique

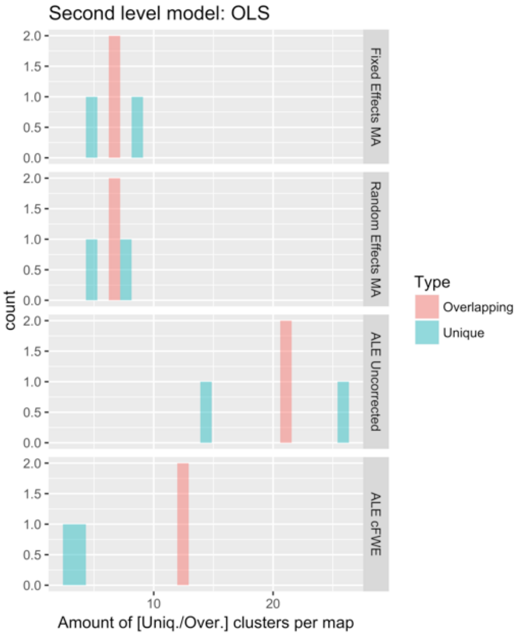
Second level model: OLS
count
Fixed Effects MA
Random Effects MA
ALE Uncorrected
ALE cFWE
Amount of [Uniq./Over.] clusters per map
Type
Overlapping
Unique

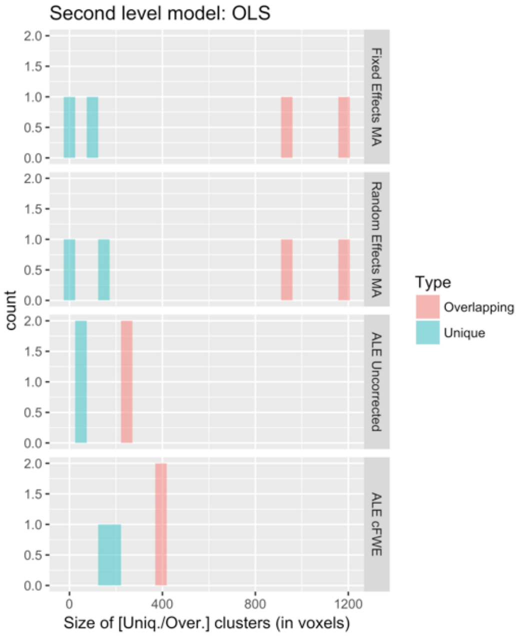
Second level model: OLS
count
Fixed Effects MA
Random Effects MA
ALE Uncorrected
ALE cFWE
Size of [Uniq./Over.] clusters (in voxels)
Type
Overlapping
Unique

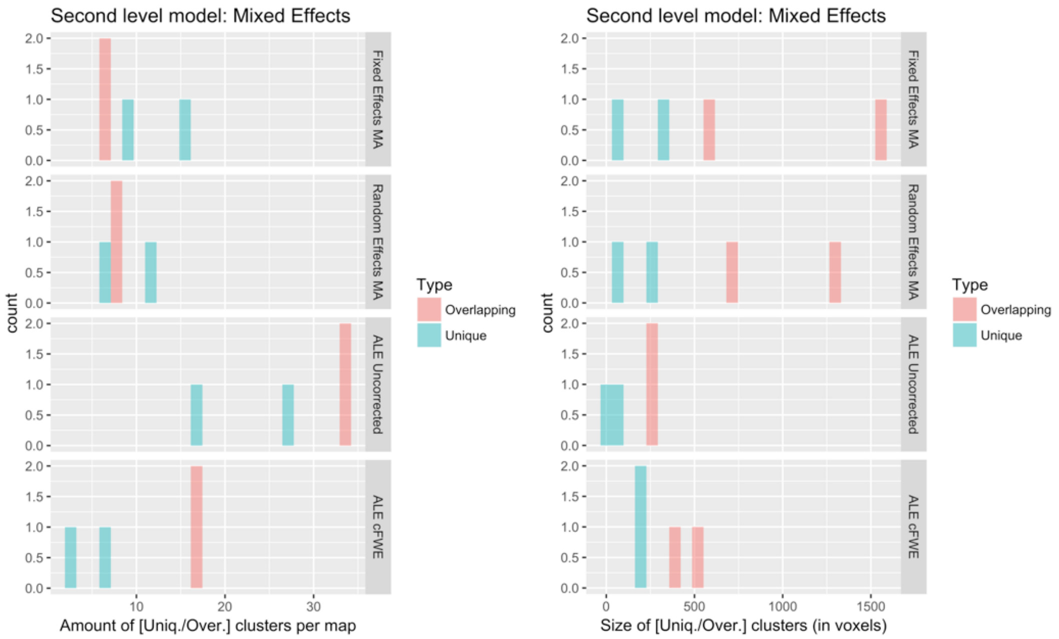
Second level model: Mixed Effects
count
Fixed Effects MA
Random Effects MA
ALE Uncorrected
ALE cFWE
Amount of [Uniq./Over.] clusters per map
Type
Overlapping
Unique
Second level model: Mixed Effects
count
Fixed Effects MA
Random Effects MA
ALE Uncorrected
ALE cFWE
Size of [Uniq./Over.] clusters (in voxels)
Type
Overlapping
Unique

## 8 Distributions of within- and between-study variability.

In the following figure, we plot the distributions of estimated within- and between-study variability in the random effects coordinate-based meta-analyses. Estimates for within-study variability are subdivided into the studies corresponding sample sizes. The x-axis is the cube root of variance, to aid visualization in the presence of large outlying values. Horizontal panes are the different group level models.

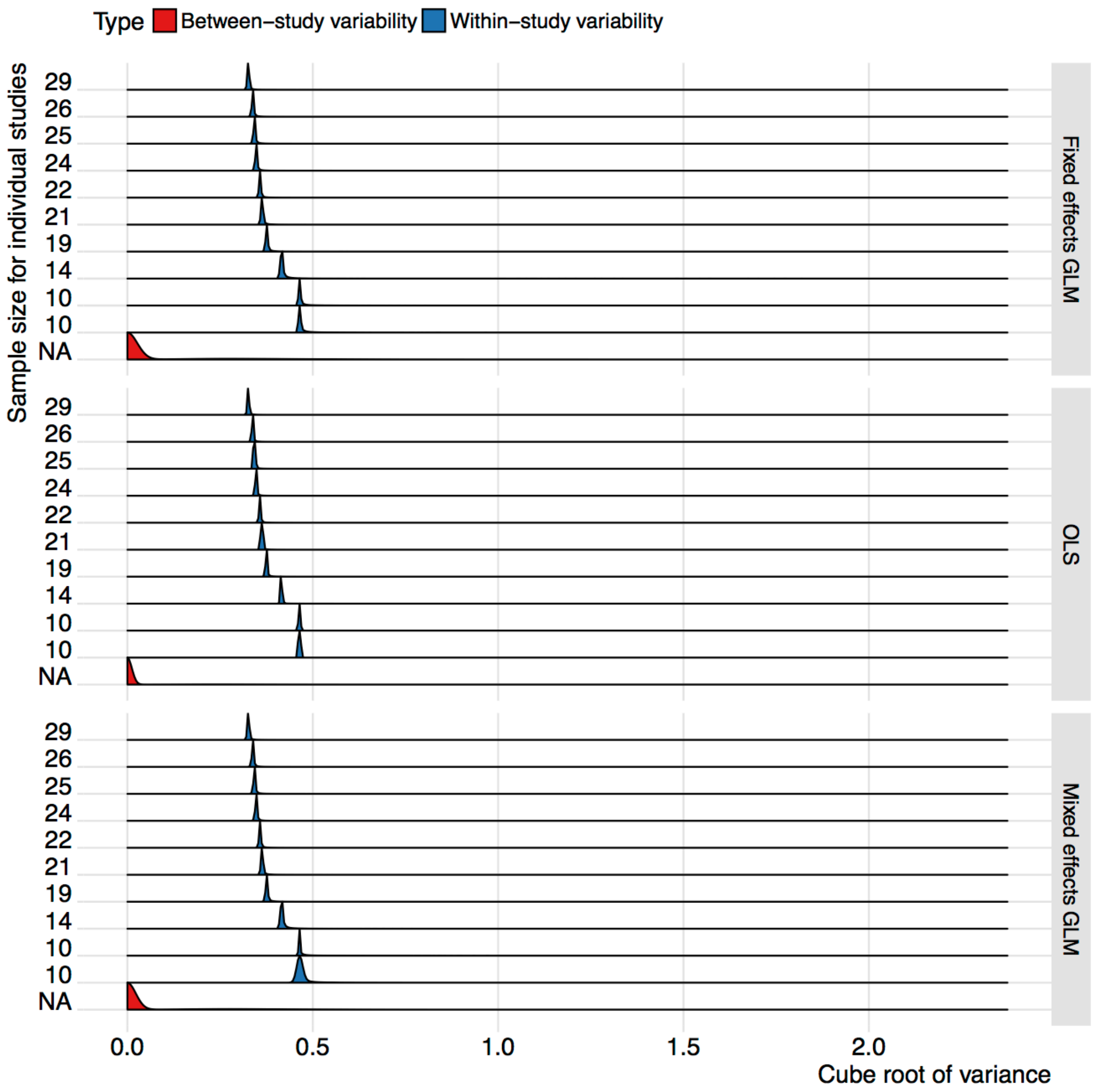

Supplement: Supplementary file 1 [file Image1.PDF]
